# From Soil to Serum: Matrix-Specific Per- and Polyfluoroalkyl Substance Accumulation and Potentially Associated Environmental Exposure Determinants in Teenagers Residing near an Industrial Hotspot

**DOI:** 10.3390/toxics14050360

**Published:** 2026-04-24

**Authors:** Jodie Buytaert, Bianca Cox, Thimo Groffen, Robin Lasters, Lieven Bervoets, Elly Den Hond, Stefan Voorspoels, Liesbeth Bruckers, Nicolas Van Larebeke, Greet Schoeters, Marcel Eens, Dries Coertjens, Ann Colles

**Affiliations:** 1 ECOSPHERE, Department of Biology, University of Antwerp, Groenenborgerlaan 171, 2020 Antwerp, Belgium; thimo.groffen@uantwerpen.be (T.G.); robin.lasters@uantwerpen.be (R.L.); lieven.bervoets@uantwerpen.be (L.B.); 2 Flemish Institute for Technological Research (VITO) Health, Boeretang 200, 2400 Mol, Belgium; bianca.cox@vito.be (B.C.); ann.colles@vito.be (A.C.); 3 Provincial Institute of Hygiene (PIH), Kronenburgstraat 45, 2000 Antwerp, Belgium; elly.denhond@provincieantwerpen.be; 4 Flemish Institute for Technological Research (VITO) GOAL, Boeretang 200, 2400 Mol, Belgium; stefan.voorspoels@vito.be; 5 Data Science Institute, University of Hasselt, Agoralaan Building D, 3590 Diepenbeek, Belgium; liesbeth.bruckers@uhasselt.be; 6 Analytical, Environmental and Geochemistry, Vrije Universiteit Brussel, Pleinlaan 2, 1050 Brussels, Belgium; nicolas.vanlarebeke@vub.be; 7 Department of Biomedical Sciences & Toxicological Centre, University of Antwerp, Universiteitsplein 1, 2610 Wilrijk, Belgium; greet.schoeters@uantwerpen.be; 8 Behavioural Ecology and Ecophysiology Group, Department of Biology, University of Antwerp, Universiteitsplein 1, 2610 Wilrijk, Belgium; marcel.eens@uantwerpen.be; 9 Centre for Research on Environmental and Social Change (CRESC), Department of Sociology, University of Antwerp, Sint-Jacobstraat 2, 2000 Antwerp, Belgium; dries.coertjens@uantwerpen.be

**Keywords:** garden, eggs, bioaccumulation, crops, rainwater, house dust, serum, per- and polyfluoroalkyl substances

## Abstract

The extensive production and use of per- and polyfluoroalkyl substances (PFAS) over recent decades have resulted in their pervasive distribution in environmental compartments worldwide. PFAS concentrations in soil and biota near fluorochemical manufacturing facilities tend to be typically higher near hotspots, which suggests that the consumption of home-produced foods near such hotspots most likely results in higher human exposure. One prominent European hotspot is located near the 3M fluorochemical production facility in Zwijndrecht (Belgium), where the relative contributions of different exposure pathways remain insufficiently characterised. This study therefore aimed to assess the PFAS concentrations and compositional profiles in serum, dwellings and gardens of teenagers residing near this hotspot. Serum samples from teenagers, along with multiple environmental matrices (i.e., soil, compost, vegetables/fruits/nuts, chicken eggs, rainwater and indoor house dust) were analysed for 21 selected PFAS. Additionally, potential determinants of PFAS occurrence and distribution across matrices were investigated using detailed questionnaire data. We found perfluorooctane sulfonic acid (PFOS) to be the predominant compound in both soil and serum, while perfluorobutanoic acid (PFBA) was most dominant in rainwater, compost, house dust and pods. Perfluorobutane sulfonic acid (PFBS) was most abundant in fruits and chicken eggs, while perfluorododecanoic acid (PFDoDA) was predominant in rooting vegetables and nuts. N-methylperfluorooctane sulfonamidoacetic acid (MePFOSAA) was the dominant compound in fruiting, stem, and leafy vegetables. These results indicate differences in accumulation pathways among the different media and/or differences in affinities of different PFAS in the matrices. Additionally, several environmental and behavioural factors were identified as determinants for PFAS in soil, compost, tree fruits, fruiting vegetables, chicken eggs and house dust, providing insight into potential drivers of exposure variability. The most important factors were related to the soil characteristics, the composting of grass and weeds, the chicken feed (i.e., bread, commercial feed), the type and frequency of ventilation and the frequency of cleaning.

## 1. Introduction

Our modern way of life results in human exposure to many chemicals that are industrially produced and used in various consumer products [1,2,3]. One group of synthetic, persistent, and toxic chemicals are per- and polyfluoroalkyl substances (PFAS). PFAS are ubiquitously present in the environment due to their long industrial production history, widespread usage in multiple consumer products, persistency and accumulation in various organisms, including humans [4]. Human exposure to some of these PFAS has already been linked with health effects [5,6,7,8], although their relative potencies are not well studied. Humans are primarily exposed to PFAS through their diet [9,10,11,12] and several PFAS have been reported in commercial and home-produced food [13]. In addition, inhalation of air and ingestion of dust may contribute significantly to the overall PFAS exposure in humans [9]. Given the associated health risks, it is important to investigate the presence of PFAS in the environment and corresponding human exposure to assess routes of exposure and possibilities for exposure reduction and remediation [8]. 

During the period from 2006 to 2012, the European Food and Safety Authority (EFSA) performed a largescale meta-analysis on the occurrence of 27 PFAS in commercial food from 13 European countries [11]. One of the main conclusions was that the contribution of dietary PFAS exposure to humans differs among food product types, as well as among PFAS compounds [11]. For example, the highest contributor to the dietary exposure of the general population in the EU for perfluorooctane sulfonic acid (PFOS) was consumption of fish and seafood, followed by fruits and fruit products, and then meat and meat products. On the other hand, the EFSA reported that fruits and fruit products, fish and other seafood, and eggs and egg products were the dominant contributors to human exposure to perfluorooctanoic acid (PFOA). Additionally, in 2020 EFSA published an updated evaluation on the risks on human health of PFAS in food [14]. Here, comparable conclusions were reached, indicating that fish, fruit and fruit products, and eggs and egg products represent the primary contributing food categories to the combined exposure to PFOA, PFNA, PFHxS and PFOS. However, studies remain limited regarding the accumulation of PFAS in non-commercial fruit and vegetable products and the human exposure through non-dietary routes of exposure (e.g., house dust).

PFAS concentrations in the environment are typically higher near hotspots [15,16,17], such as fluorochemical industry and fire training facilities, which may imply that consumption of self-cultivated food can result in higher human exposure in these areas [18]. One well-known example of a PFAS hotspot is the contamination surrounding the 3M fluorochemical manufacturing facility in Zwijndrecht, Belgium. Although PFAS concentrations in this area have been investigated both in plant and animal species [19], as well as in soil, and human serum samples [8], still little is known about the PFAS distribution within a terrestrial environment as well as possible human exposure pathways through environmental media. Additionally, local factors in private gardens have been shown to be associated with PFAS concentrations found in self-cultivated food and in the resulting human exposure. However, little is known about which determinants may influence PFAS concentrations in other environmental matrices and, consequently, the exposure of teenagers living near a hotspot. Therefore, this study aimed to characterise the PFAS concentrations and compositional profiles present in serum, dwellings and gardens of teenagers living within a 5 km radius of the 3M facility in Zwijndrecht, Belgium. We measured 21 target PFAS in serum of teenagers as well as in different environmental media (soil, compost, vegetables/fruits/nuts, chicken eggs, rainwater and house dust), enabling the investigation of the differences and similarities across these matrices. A secondary aim was the identification of possible determinants that may explain the PFAS accumulation profiles in these matrices. In contrast to previous single-medium studies, this research is the first to systematically investigate the multimedia PFAS exposure pathway from the environment to humans within the same adolescent population and to assess the potential influence of local ‘no-regret’ measures.

## 2. Materials and Methods

### 2.1. Study Area and Study Population

**Figure 1:**
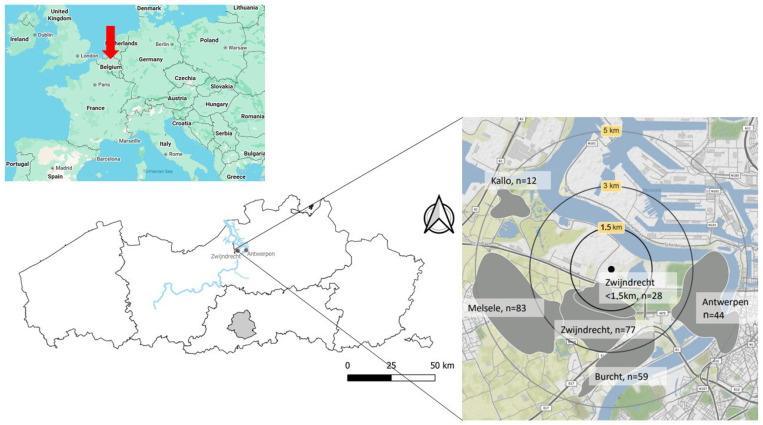
Map of the study area situated in Europe and Flanders, Belgium. The study area consisted of a circular area with a radius of 5 km surrounding the fluorochemical manufacturing facility in Zwijndrecht, Flanders (Flemish part of Belgium), and indicating the 6 sampling zones corresponding to different cities and the number of participants living in these zones. The two subset figures were made using Google Maps.

The study area consisted of a circular area with a radius of 5 km surrounding the 3M facility in Zwijndrecht, Belgium (Figure 1). This area is a well-studied PFAS contaminated site linked to the facility’s activities since 1971 [18,20,21,22,23]. Due to the widespread PFAS contamination in this area, the local government has implemented several “no-regret” measures in 2021 to reduce PFAS-associated exposure to local contaminated media. These measures are not legally binding and are recommendations. An example is the advice not to consume home-produced chicken eggs. These no-regret measures apply throughout the study area and vary in stringency depending on the distance of the dwelling from the point source, with stricter measures closer to the facility. The north and northwestern part of this area consists mainly of industrial sites, while the rest of the study area consists of urban and agricultural areas. In this area, participants born in 2006, 2007, 2008 or 2009 were recruited through invitation letters. Participants were selected based on predefined criteria and were included if; (i) both the teenager and the parents gave permission to participate and signed the informed consent form (ICF); (ii) the teenager had lived in the research area for at least the past 5 years; and (iii) both the teenager and the parents were able to fill in an extensive questionnaire in Dutch. In total, 303 participants were recruited. Each participant received four questionnaires: a general questionnaire for the parents about housing history, characteristics of the home and garden and use of consumer products; a general questionnaire for the teenager regarding his/her country of origin, habits and use of consumer products; a questionnaire about consumption of locally grown food that the teenager had to fill out with the help of parents and finally; and a questionnaire about the vegetable garden and/or chicken stock at the residence, filled out by the parents. These questionnaires were necessary to assess the general characteristics of the study population and to identify the potential determinants of PFAS concentrations in the environmental matrices. For these determinants, only those with at least six participants in each response category were included in further analyses. Here, we were able to analyse determinants for the following matrices: vegetable garden soil, compost, tree fruits, fruiting vegetables, chicken eggs and house dust. The different determinants analysed can be found in Tables S1–S3 of the Supplementary Information (SI).

### 2.2. Sampling Method

The sampling was carried out from July to September 2022, and was in accordance with the guidelines prepared by The Public Waste Agency of Flanders (OVAM) [24]. The soil samples from the vegetable garden were taken from the top 20 cm, as this is the cultivated layer. Additionally, since plant roots often extend deeper, sampling this depth provides a representative picture of what plants can effectively absorb. For the soil of the chicken enclosure, the top 10 cm was sampled, as chickens mainly forage and scratch in the surface layer. Therefore, any contaminants present in this layer are the most relevant for uptake by the chickens. During this sampling period, PFAS manufacturing activities at the facility were suspended, which may have influenced environmental PFAS concentrations. In addition to soil, rainwater and compost samples were also collected from the private gardens of the study participants, also in accordance with the guidelines of OVAM. Rainwater samples were collected from barrels, with the sampling methodology varying depending on the barrel type (see SI, text 1C). Different types of vegetables, fruits, nuts and chicken eggs were sampled and were classified as shown in Table S4. These food samples were collected using an opportunistic sampling strategy, i.e., samples were taken of fruits, vegetables and nuts that were already present in the private gardens. House dust samples were collected by the participants using a scraping method. For this, participants received a plastic scraper, a piece of aluminium foil and a plastic bag. Participants collected house dust by scraping an uncleaned surface onto aluminium foil, which was subsequently folded and placed in a storage bag. Serum sampling took place at central locations in the study area between 28 June and 31 August 2022 and was carried out by an experienced team of nurses. A peripheral blood sample of 39 mL was collected and were immediately centrifuged for 20 min at 3500 rpm at the sampling location. Serum was aliquoted in a cryotube, kept at 4 °C, transported on dry ice and was stored at −80 °C within 12 h, until analysis. The remaining serum volumes were used to measure biomarkers of effect [8] and were stored as biobank samples. More detailed information regarding the sampling method for each matrix can be found in the SI (Text 1a–g). Sample sizes per matrix can be found in Table 1.

### 2.3. PFAS Extraction and Measurement

Different extraction protocols were used depending on the matrix. All abiotic samples and serum were extracted using methanol as a solvent followed by solid-phase extraction (SPE). All remaining biotic samples were extracted using acetonitrile (ACN) as a solvent followed by a clean-up step using graphitized carbon absorbent. Serum samples were analysed using an in-house developed procedure. All samples were spiked with an internal standard mixture; for fruits, vegetables, nuts and chicken eggs, samples were spiked using 10 ng of MPFAC-MXA (Wellington Laboratories, Guelph, ON, Canada); for soils, compost, rainwater, house dust and serum samples, samples were spiked using 4 ng of a mixture of 10 mass-labelled internal standards (^13^C_4_-PFBA, ^13^C_4_-PFPeA, ^13^C_2_-PFHxA, ^13^C_4_-PFOA, ^13^C_5_-PFNA, ^13^C_2_-PFDA, ^13^C_2_-PFUnA, ^13^C_2_-PFDoA, ^18^O-PFHxS, ^13^C_4_-PFOS; Wellington Laboratories, Guelph, ON, Canada). More detailed information regarding extraction procedures per matrix is given in the SI (text 2a). A total of 43 target PFAS, of which for seven of these compounds (i.e., PFOA, perfluorohexanesulfonic acid (PFHxS), PFOS, N-methylperfluorooctanesulfonamide (MePFOSA), N-ethylperfluorooctanesulfonamide (EtPFOSA), N-methylperfluorooctanesulfonamido acetic acid (MePFOSAA) and N-ethylperfluorooctanesulfonamido acetic acid (EtPFOSAA)), both the ‘linear’ and ‘linear + branched’ forms were measured using ultra-performance liquid chromatography-coupled tandem mass spectrometry (UPLC-MS/MS) (Table S5). First, all 43 PFAS were analysed in a selection of 50 blood serum and 50 soil samples distributed across the whole study area. Based on these results, a final selection of 21 PFAS that were quantifiable (above the Limit of Quantification (LOQ)) in at least 10% of the screened sampled was retained for analysis in all remaining samples. Of these 21 PFAS, five were measured in both their ‘linear’ and ‘linear + branched’ forms (Table S5). More detailed information regarding the UPLC-MS/MS analysis per matrix is given in the SI (text 2b, Table S6).

### 2.4. Quality Control and Assurance

For fruits, vegetables, nuts and chicken eggs the following QC measures were in place. As instrumental blanks, 100% ACN was injected on regular basis to limit cross-over contamination between samples, as well as between samples and procedural blanks. Per batch of 15–20 samples, one procedural blank (i.e., 10 mL of ACN) was used to correct for possible contamination occurring during extraction or analysis. Procedural blank values are shown in Table S7. We verified whether PFAS concentrations were in the linear range of the calibration curves and diluted samples when necessary. For soils, compost, house dust and serum samples the following QC measures were in place. Each measurement series consisted of 20 samples and was accompanied by the necessary quality control measurements, such as control standards (measurement standards for calibration and integration standards for evaluating the branched isomers), solvent blanks, procedural blanks, a duplicate sample and a spiked reference sample (when possible, conditions apply for house dust due to limited variability). Recovery rates of 70–130% were considered acceptable. For duplicate samples, a deviation of 30% was flagged. All LOQs are based on a signal-to-noise ratio of 10 and was validated in real matrix samples following spikes at LOQ mass fraction. The methods for soil, compost, house dust and serum samples are validated within international ring-test studies and all except for house dust are BELAC ISO 17025 accredited (BELAC 045-TEST). All LOQ values can be found in Table S8.

### 2.5. Soil Characteristics

From the soil samples, moisture content, clay content, total organic carbon (TOC) content and pH were determined. Moisture content was determined by drying the samples in an oven at 105 °C until the weight remained stable and dry matter was then expressed as a percentage [25]. Clay content was determined through a texture analysis in which the grainsize fractions of the mineral soil are separated (sand, loam and clay) and quantified. This analysis was carried out on the fine soil fraction (<2 mm), after separation from the coarse elements [26]. TOC content was determined by using the direct method in which the carbonates present in the sample are removed by treating the sample with hydrochloric acid [27]. At last, pH was determined by means of a potential measurement using 2 calibrated electrodes [28]. Further details on the analysis can be found in the Supplementary Information.

### 2.6. Statistical Analysis

For PFAS with a detection frequency of at least 30% in a given matrix and with at least 10 values >LOQ, concentrations <LOQ were imputed using single random imputation from a censored lognormal distribution within each matrix (MLE substitution). Censored values were imputed by randomly sampling from the fitted lognormal distribution in the range 0–LOQ. To assess the sensitivity of this MLE method, the compositional profiles of chicken eggs were determined in four different ways, i.e., by using MLE substitution, LOQ/2 substitution, LOQ = 0 substitution, and by only using concentrations above LOQ. All four profiles (Figure S1) were highly similar, reinforcing that the conclusions drawn when using MLE substation remain robust. Because the environmental samples were collected on private properties of citizens and due to the small sample sizes for some matrices, we followed the principles of the Research Data Centres of the Federal Statistical Office and the Statistical offices of the Federal States (RDC) in Germany regarding data protection [29] In line with these principles, the protection of personal data was ensured in the reporting of percentiles: the p50 (median) was reported only when N ≥ 6, and the P25-P75 percentiles were reported only when N ≥ 12. For variables for which no imputations could be performed, the compound was dichotomised into <LOQ versus ≥LOQ, and this binary variable was used in further analyses. For each matrix, the PFAS compositional profile was determined by calculating the median concentration of each compound, adjusted for its molar mass, and expressed as a proportion of the total median PFAS concentration (adjusted for the molar masses). Only compounds present in >50% of the samples were included in the profiles. Additionally, to help visualise the differences in PFAS compositional profiles, a hierarchical clustering analysis was performed on scaled molar-adjusted median PFAS profiles across matrices using Euclidean distance and Ward’s linkage method (Ward.D2), and results were visualised using a heatmap with an accompanying dendrogram. We opted for this method, since a principle component analysis (PCA) requires that all observations are measured on the same set of variables, resulting in a complete rectangular data matrix. In the dataset of this study, PFAS detection patterns differ substantially between matrices, with several PFAS detected consistently in some matrices, but detected too infrequently or not at all in others. As a result, a common set of variables suitable for PCA could not be established. Associations between environmental PFAS concentrations and potential determinants obtained from questionnaires were assessed through unadjusted regression analyses. We used separate models for each PFAS compound, matrix, and determinant. PFAS with a detection frequency of at least 60% in a given matrix were analysed as continuous outcomes using linear regression, whereas compounds below this threshold were dichotomised (<LOQ vs. ≥LOQ) and analysed using logistic regression models. Continuous PFAS concentrations were natural log-transformed to adjust for their skewed distributions. Estimates from the linear models are expressed as geometric mean ratios (GMRs), which quantify the relative change in the geometric mean PFAS concentrations associated with a given determinant. For categorical determinants, GMRs compare each category with a reference category, whereas for continuous determinants they represent the change in geometric mean concentrations per interquartile range (IQR) increase in the determinant. These GMRs are expressed as percentages converted to decimal form. Estimates from logistic models are presented as odds ratios. The significance level for all tests was set at 0.05. Because a large number of determinant tests were performed, *p*-values were additionally adjusted within each matrix using the Benjamini–Hochberg false discovery rate (FDR) procedure. Adjusted *p*-values are provided in the Supplementary Material for reference, whereas interpretation in the main text is based on the unadjusted analyses due to the exploratory nature and limited sample sizes. Analyses were performed using the glm procedure in SAS version 9.4 (SAS Institute, Cary, NC, USA).

## 3. Results

### 3.1. Description of Study Population

Most participants were 12.5–14.5 years old (41%), while the age groups of 14.5–15.5 years (35%) and 15.5–17 years (24%) were somewhat smaller. The study population contained an almost equal proportion of girls (51%) and boys (49%). According to the questionnaires, the majority of the study population (67%) had no chickens in their private garden and had never consumed locally produced chicken eggs, 10% had already stopped eating home-grown chicken eggs before the announcement of the local no-regret measures, 14% stopped after the announcement, and 8% continued to eat home-produced chicken eggs. The majority of the participants did not have a vegetable garden with vegetables or fruits present (64%). Among those who did, most grew their crops directly in the soil (25%), while a smaller proportion used planters (12%). Moreover, respectively 52% and 53% had never eaten small fruits and tree fruits from their own private garden. Furthermore, 53% never consumed vegetables from their own garden, 34% had not consumed any in the past three months, 8% consumed them monthly, and 5% consumed them weekly. A compost heap or barrel was owned by 25% of the participants, of whom 6% and 10% reported using it in their vegetable garden. Additionally, 14% of the participants currently owned a groundwater well, 6% had owned one in the past and 80% had never owned one. All participants who had access to such a groundwater well reported using it on a regular basis, e.g., washing and cooking vegetables, preparing hot beverages, irrigation, hygiene, and dishwashing.

### 3.2. PFAS Concentrations and Compositional Profiles in the Different Matrices

**Figure 2:**
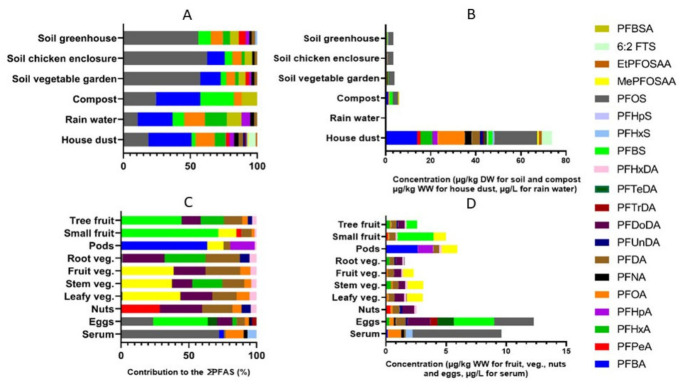
(**A**) (abiotic matrices) and (**C**) (biotic matrices): percentage contribution of individual PFAS compounds, to the total ∑PFAS concentration (based on the molecular weight-corrected median concentrations) for compounds with a detection frequency of at least 50% in that specific matrix, with soil and compost concentrations expressed in µg/kg DW, house dust, fruits, vegetables, nuts and eggs expressed in µg/kg WW, rainwater concentrations expressed in µg/L and serum concentrations expressed in µg/L, (**B**) (abiotic matrices) and (**D**) (biotic matrices): absolute median concentrations of PFAS compounds, for compounds with a detection frequency of at least 50% in that specific matrix. For all compounds for which both linear and linear+branched forms were analysed, we have used the linear+branched concentrations for these figures.

Median PFAS concentrations (P25–P75) and detection frequencies in analysed matrices can be found in Tables S9 and S10. The median concentrations are also displayed in Figure 2B,D. Molecular weight-corrected PFAS compositional profiles in abiotic and biotic matrices are displayed in Figure 2A and Figure 2C, respectively.

**Figure 3:**
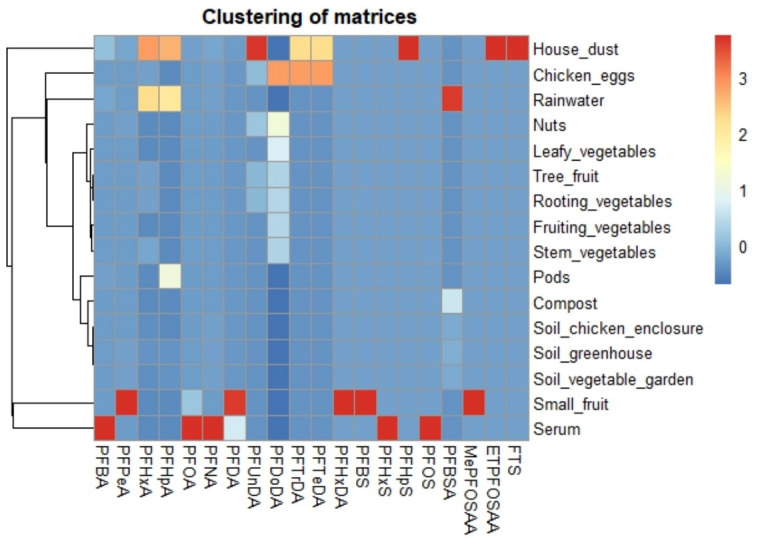
Hierarchical clustering of the molar-adjusted median PFAS profiles across matrices presented as a heatmap with accompanying dendrogram.

The PFAS profiles and absolute concentrations of the different soil types were similar, as reflected by their clustering in the dendrogram (Figure 3), with ∑PFAS concentrations of 3.56 µg/kg dry weight (DW) for both the chicken enclosure soil and greenhouse soil, and 4.00 µg/kg DW for soil from the vegetable garden. The profiles consisted mostly of long-chained PFAS, of which PFOS_linear+branched_ was the dominant compound. Besides long-chained PFAS, also short-chained PFAS were present in the different soil types, from which perfluorobutanoic acid (PFBA) and perfluorobutanesulfonic acid (PFBS) were the most dominant. Remarkably, PFBA was not present in soil from the greenhouse. Compared to soil, PFAS concentrations in compost were slightly higher (∑PFAS concentration of 5.39 µg/kg); however, the PFAS profile was less diverse, with only five compounds detected in compost versus an average of nine in soil. Here, short-chained PFAS (i.e., PFBA and PFBS) and PFOS were the main compounds. Furthermore, since it is difficult to compare concentrations between liquid and solid matrices, we assumed 1 L ≈ 1 kg. Under this assumption, the concentrations measured in rainwater were of an order of magnitude lower compared to concentrations found in all other matrices. In rainwater, mostly short-chained PFAS (especially perfluorocarboxylic acids (PFCAs)) were present, of which PFBA was the most dominant compound. Finally, among all collected matrices, house dust had the highest number of detected PFAS and the highest absolute concentrations, with levels up to 18 times higher than those in soil, 6 times higher than in eggs, and up to 48 times higher than in fruits and vegetables (Figure 2A,B). Here, 27 different compounds were detected, with the largest contributions to the profile for PFBA, PFOS_linear+branched_ and PFOA_linear+branched_.

Of all biotic matrices, chicken eggs clearly exhibited the highest ∑PFAS concentrations, with PFBS as the most dominant compound (40% of total profile). Within the fruits, vegetables and nuts, both the pods and small fruits showed the highest concentrations and the PFAS profiles were overall dominated by PFCAs (Figure 2C), either short- or long chained, depending on the matrix. Long-chained PFAS were most dominant in tree fruits, leafy, stem, fruiting and rooting vegetables and nuts, whilst short-chained PFAS were most dominant in small fruits and pods. Remarkably, the profile of the leafy and fruiting vegetables did not contain any short-chained compounds. Whilst PFOS_linear+branched_ was most dominant in the different soil types, it was not present in the profiles of fruits, vegetables and nuts, except for a very small contribution in leafy and rooting vegetables. Furthermore, the fruit and vegetable categories exhibit similar profiles, as evidenced by their clustering within the dendrogram (Figure 3).

In serum, PFOS_linear+branched_ was the most dominant compound. PFBS dominated the egg profile despite having only a small contribution in soils. Besides PFCAs and perfluorosulfonic acids (PFSAs), the precursor compound MePFOSAA_linear+branched_ had a large contribution to the profiles of small fruits, leafy vegetables, stem vegetables, fruiting vegetables and pods. This compound was not present in the PFAS profile of any of the abiotic samples, nor in tree fruits, root vegetables or nuts. Moreover, whilst perfluorobutane sulfonamide (PFBSA) was present in all abiotic samples (except house dust), it was not present in any of the biotic PFAS profiles. Finally, median ∑PFAS concentrations in serum were 9.5 µg/L, of which PFOS and PFOA were the most dominant compounds.

### 3.3. Determinants of PFAS in Different Environmental Matrices

All variables tested as potential determinants of PFAS concentrations in vegetable garden soil, chicken enclosure soil, compost, tree fruits, fruiting vegetables, chicken eggs and house dust are listed in Tables S1–S3 of the Supplementary Information. Given the exploratory nature of the study and the limited sample sizes for several matrices, the determinant analyses were limited to unadjusted univariable regression models because multivariable regression requires an adequate ratio of observations to parameters. Several matrices in our study contained fewer observation than required to fit stable multivariable models, which would lead to overfitting, inflated variances, and non-interpretable estimates. Therefore, multivariable modelling was not appropriate and univariate exploratory analyses were used instead. Additionally, Section 3.3.1, Section 3.3.2, Section 3.3.3 and Section 3.3.4 present the associations that were significant in the unadjusted analyses, while FDR-adjusted *p*-values are provided in the Supplementary Material. After FDR correction, only a subset of associations remained statistically significant; therefore, results should be interpreted cautiously, with emphasis placed on overall patterns rather than on individual *p*-values.

#### 3.3.1. Soil and Compost

**Figure 4:**
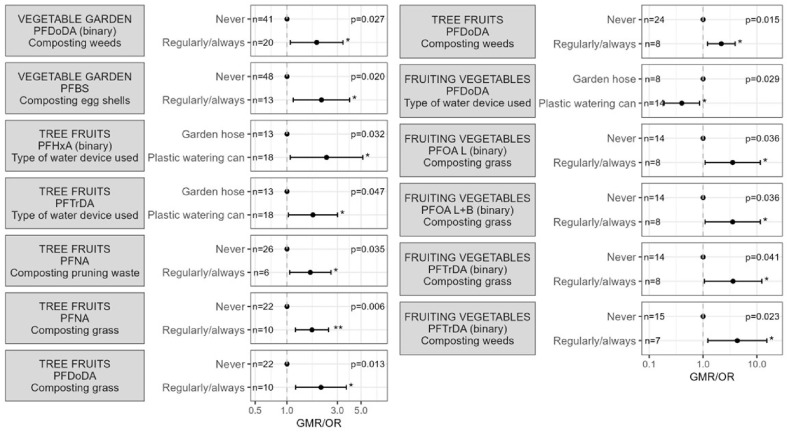
Significant determinants for PFAS concentrations in vegetable garden soil, chicken enclosure soil, compost, tree fruits and fruiting vegetables. The estimates (with 95% confidence intervals) represent the geometric mean ratio (GMR; for continuous PFAS) or odds ratio (OR; for binary PFAS) for each category of the determinant compared to the reference category (the category with GMR or OR = 1). The overall *p*-value is presented in the upper right corner, category-level *p*-values are presented next to the estimates as: * *p* < 0.05; ** *p* < 0.01.

In addition to the questionnaire-based determinants, soil characteristics were investigated. Significant results of the univariate analysis can be found in Tables S11 and S12, and are visualised in Figure 4, except for the soil characteristic results, which are not included in Figure 3 but are fully reported in the Supplementary tables. For the vegetable garden soil, significant positive associations were found between the percentage of total organic carbon and the concentrations of perfluoropentanoic acid (PFPeA), perfluorohexanoic acid (PFHxA), perfluoroheptanoic acid (PFHpA), PFOA_linear+branched_, perfluorododecanoic acid (PFDoDA), PFBS and PFBSA. In chicken enclosure soil, total organic carbon was positively associated with PFOA, PFOA_linear+branched_, perfluorononanoic acid (PFNA), perfluorodecanoic acid (PFDA), PFHpA, perfluoroundecanoic acid (PFUnDA) and PFDoDA. Additionally, PFBA, PFPeA, PFHxA, PFHpA, PFOA, PFOA_linear+branched_, PFBS and PFBSA concentrations in the soil of the vegetable garden were also significantly higher when there was a higher contribution of particles < 2 µm in the soil. For the soil of the chicken enclosure, no significant associations were found with the contribution of particles < 2 µm. Several significant negative associations were found between the soil pH and PFAS concentrations of the vegetable garden (PFOS_linear_ and PFOS_linear+branched_) and chicken enclosure soil (PFNA, PFDA, PFUnDA, PFOS_linear_, PFOS_linear+branched_ and PFBSA). We also found a significant negative association between PFBS concentrations and the percentage of dry matter in the soil of the vegetable garden, while no such associations were observed for other compounds or for chicken enclosure soil.

Irrigation of the vegetable garden with either ground, tap, or rainwater was not associated with the soil PFAS concentrations, nor was the use of either a garden hose or a plastic watering can. The composition of the compost was associated with PFDoDA concentrations in the soil of the vegetable garden, with significantly higher concentrations observed when composted weeds were used. Furthermore, PFBS concentrations were significantly higher in the soil of the vegetable garden when participants reported throwing eggshells into their compost heaps. Composting of grass clippings, pruning waste, and fruit and vegetable scraps were not associated with PFAS concentrations in the soil of the vegetable garden.

#### 3.3.2. Tree Fruits and Fruiting Vegetables

Significant results of the univariate analysis can be found in Table S13 and are visualised in Figure 4. Firstly, PFAS concentrations in tree fruits and fruiting vegetables were associated with three determinants concerning composting: composting of grass clippings (PFNA and PFDoDA), weeds (PFDoDA), and pruning waste (PFNA), with significantly higher concentrations when these items were composted (Figure 3). The composting of food scraps and eggshells, on the other hand, did not appear to be associated with PFAS concentrations in tree fruits and fruiting vegetables. Furthermore, the type of watering device seemed to be a potential determinant for PFAS in both tree fruits and fruiting vegetables. PFTrDA and PFHxA concentrations in tree fruits were significantly higher when using a plastic watering can compared to a garden hose, but the opposite was observed for PFDoDA in fruiting vegetables.

#### 3.3.3. Chicken Eggs

**Figure 5:**
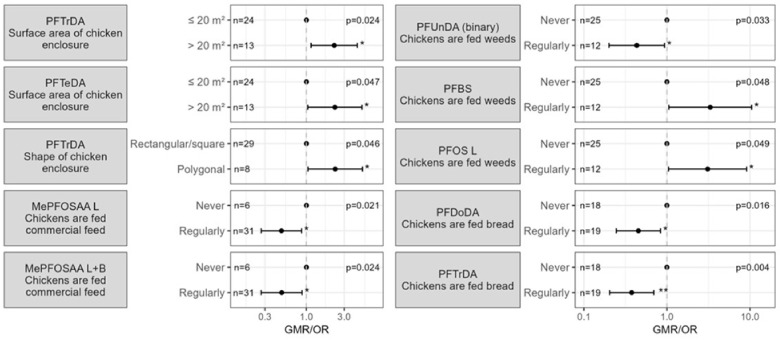
Significant determinants for PFAS concentrations in chicken eggs. The estimates (with 95% confidence intervals) represent the geometric mean ratio (GMR; for continuous PFAS) or odds ratio (OR; for binary PFAS) for each category of the determinant compared to the reference category (the category with GMR or OR = 1). The overall *p*-value is presented in the upper right corner, category-level *p*-values are presented next to the estimates as: * *p* < 0.05; ** *p* < 0.01.

Significant results of the univariate analysis can be found in Table S14 and are visualised in Figure 5. Firstly, some properties of the free-range space, such as the shape and the size, seemed to be associated with the PFAS concentrations in chicken eggs. Both perfluorotridecanoic acid (PFTrDA) and perfluorotetradecanoic acid (PFTeDA) concentrations in eggs from chickens living in a larger (>20 m^2^) enclosure were significantly higher compared to concentrations in eggs from chickens living in smaller enclosures. We further found that PFTrDA concentrations in eggs from chickens living in a polygonal enclosure were significantly higher compared to those in eggs from chickens living in a square or rectangular enclosure. Additionally, chickens regularly fed commercial feed had eggs with significantly lower MePFOSAA and MePFOSAA_linear+branched_ concentrations compared to chickens that were never fed commercial feed. Chickens regularly or often fed bread produced eggs with significantly lower PFDoDA and PFTrDA concentrations compared to chickens that never consumed bread. In contrast, egg concentrations of PFBS, PFUnDA and PFOS_linear_ were significantly higher when chickens were regularly fed weeds from their own garden. The use of grass and food scraps as chicken feed did not appear to be associated with PFAS concentrations in eggs.

#### 3.3.4. House Dust

**Figure 6:**
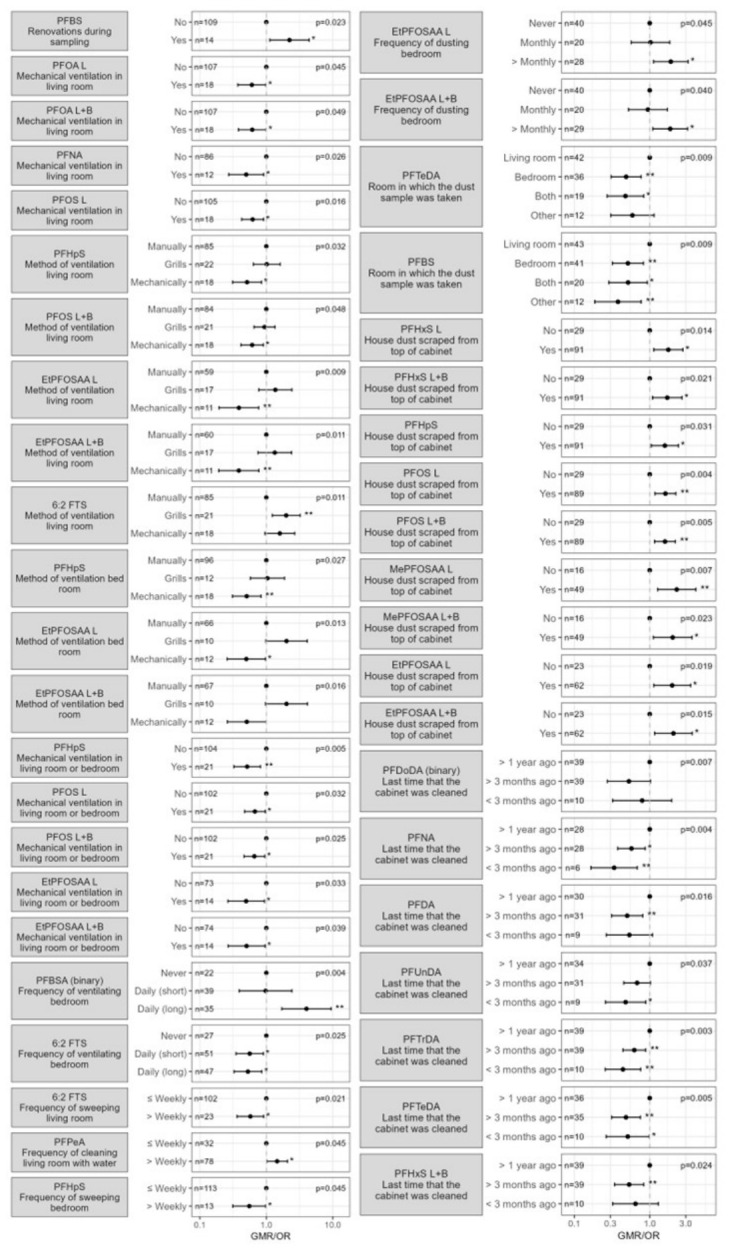
Significant determinants for PFAS concentrations in house dust, part one. The estimates (with 95% confidence intervals) represent the geometric mean ratio (GMR; for continuous PFAS) or odds ratio (OR; for binary PFAS) for each category compared to the reference category of the determinant (the category with GMR or OR = 1). The overall *p*-value is presented in the upper right corner, category-level *p*-values are presented next to the estimates as: * *p* < 0.05; ** *p* < 0.01.

Significant results of the univariate analysis can be found in Table S15 and are visualised in Figure 6. Firstly, home renovations during the sampling period were associated with higher concentrations of PFBS in house dust. In contrast, the use of a mechanical ventilation system in one of both sampled rooms during the sampling period was associated with lower concentrations of PFOA_linear_, PFOA_linear+branched_, PFNA, perfluoroheptanesulfonic acid (PFHpS), PFOS_linear_, PFOS_linear+branched_, EtPFOSAA_linear_ and EtPFOSAA_linear+branched_ in house dust. Mechanical ventilation also resulted in lower PFHpS, PFOS_linear+branched_, EtPFOSAA_linear_, EtPFOSAA_linear+branched_ concentrations compared to manual ventilation, whilst 6:2 fluorotelomer sulfonic acid (6:2 FTS) concentrations were higher with mechanical ventilation. 6:2 FTS concentrations in house dust were significantly lower in homes where bedrooms were ventilated daily (short or long periods) compared to bedrooms that were never ventilated. In contrast, PFBSA concentrations in house dust were significantly higher when bedrooms were ventilated daily for longer durations. Furthermore, sweeping the living room or bedroom at least weekly was associated with lower concentrations of 6:2 FTS and PFHpS in house dust compared to sweeping less than weekly. Cleaning the living room with water at least weekly was associated with higher concentrations of PFPeA in the samples compared to cleaning less than weekly. EtPFOSAA_linear_ and EtPFOSAA_linear+branched_ concentrations in house dust were significantly higher when the bedroom was dusted multiple times per month compared to never. Vacuum-cleaning and mopping did not appear to be associated with PFAS concentrations in house dust. As for the type of room sampled, house dust samples coming from either the bedroom, both living and bedroom or another room showed significantly lower levels of PFTeDA and PFBS compared to samples collected exclusively in the living room. Samples collected on a closet or cupboard had significantly higher concentrations of PFHxS_linear,_ PFHxS_linear+branched_, PFHpS, PFOS_linear_, PFOS_linear+branched_, MePFOSAA_linear,_ MePFOSAA_linear+branched_, EtPFOSAA_linear,_ and EtPFOSAA_linear+branched_ compared to samples collected on other surfaces. Samples collected from closets or cupboards that had been cleaned within the last three months or the last year showed lower concentrations of PFNA, PFDA, PFUnDA, PFDoDA, PFTrDA, PFTeDA, and PFHxS_linear+branched_ compared with those collected from closets or cupboards cleaned longer ago.

## 4. Discussion

### 4.1. PFAS Concentrations and Compositional Profiles in the Different Matrices

#### 4.1.1. Abiotic Matrices

The PFAS concentrations found in the different soil types were similar to those found in previous studies performed in private gardens within the same area [30,31]. The high presence of PFOS_linear+branched_ in all soil types can be explained by its relatively large adsorption affinity for soil surface layers, its persistent nature, and its relatively large production volume by 3M before the voluntary phase-out in 2002 [32]. Additionally, it is known that some precursor compounds can transform into PFOS through biodegradation, which can further explain the high prevalence of this compound in soil. PFAS, particularly long chain PFAS, are known to bind to soil organic matter (SOM) [33]. This might explain the presence of PFOA_linear+branched_, PFNA and PFDA in all of the soil types analysed, but out of these PFAS, only for PFOA_linear+branched_ a significant positive association was found with the total organic carbon in soil. Compared to soil, compost showed a more dominant profile of the shorter chain compounds and Sivaram, et al. [34] reported similar contributions of short-chain PFAS in compost. This might also explain the more frequent detection of short-chain PFAS in greenhouse and vegetable garden soils compared to chicken enclosure soils, as compost is more frequently applied to the former. The dominance of short-chain PFAS in compost may be linked to the relatively large input of vegetable food scraps, plant yard waste, and food-packaging materials, which are known to contain primarily short-chain PFAS [35,36]. These findings reinforce the rationale behind the current no-regret measures advising residents not to use homemade compost within a radius of 1.5 km of the factory. The high percentage of short-chain PFAS detected in the rainwater was expected. Compared to long-chain PFAS, short-chain homologues are more hydrophilic, which explains their dominant distribution in polar media such as rainwater [37]. Furthermore, short-chain PFAS are more likely to be deposited on particles in the atmosphere, and can then be deposited through the process of wet atmospheric deposition into rainwater [38]. Additionally, more PFCAs were detected in the rainwater, compared to PFSAs, which might also be due to their higher water solubility [39]. The PFAS concentrations in rainwater observed in the present study can be considered as an average value over a longer time period, because samples were collected from a rain barrel. This sampling approach reduces the risk of overestimating concentrations, since it has been shown that the first millilitres of rain contain the highest PFAS concentrations, and that concentrations decrease with more rainfall occurring. In addition, as samples were collected from a barrel, some contamination may have originated from roof runoff, with water flowing over the roof surface and being conveyed through a downpipe into the barrel. The concentrations and compounds found in the present study were similar to those in the study of Lasters, Groffen, Eens and Bervoets [19], which was carried out in the same study area. Results of the present study (∑PFAS = 32.6 ng/L) were also comparable to those reported for northern Germany (1.6–48.6 ng/L), where no clear point source is present [40]. Furthermore, the concentrations found in rainwater in our study were also of the same order of magnitude as those reported in other international studies [39,41,42]. For example, the sum of PFCA concentrations in the present study (17.9 ng/L) was similar to the range reported by, where total PFCA concentrations varied from 1.40 ng/L (India) to 18.1 ng/L (Tsukuba, Japan). In contrast, the average PFSA concentration in rainwater the present study (3.4 ng/L) was higher than those reported by, who observed average PFOS concentrations ranging from 0.04 ng/L (India) to 0.81 ng/L (Kawaguchi, Japan). This indicates spatial differences in rainwater concentrations, likely driven by differences in emission sources and the amount of PFAS present in ambient air.

The PFAS profiles of the soil matrices were similar to those of the rainwater samples (Figure 2A), likely reflecting of PFAS through passive deposition and, for vegetable garden and greenhouse soil, through irrigation with rainwater [43,44]. However, some PFAS detected in rainwater were absent in specific soil types and vice versa, suggesting that some soil types may be less affected by rainwater (e.g., greenhouse soils that are partially or fully protected by roofing, which may explain the absence of PFBA in greenhouse soil), that some compounds may leach out, or that other sources may be more relevant contributors to PFAS contamination.

PFAS concentrations in house dust of the present study were similar to those found in house dust samples in Catalonia, Spain [45] and even lower compared to the study of Fraser, et al. [46] who analysed house dust samples from Boston, USA. The dust in this study was collected by the participants by scraping house dust of interior surfaces that were not cleaned for some time. This sampling method could have introduced variability due to (i) differences in types of surfaces sampled, (ii) the age of the collected dust and (iii) the pressure applied on the scraper, which could cause fragments of surface material to be included in the sample. In our study, the activities of the PFAS production plant were set on hold during the period of house dust collection, which could have had an impact on the concentrations and composition of the collected house dust. The rich PFAS-profile of the house dust reflects the importance of both outdoor and indoor sources. Several types of PFAS have been identified in building materials (e.g., composite wood, insulation and sealants), interior finishings (e.g., carpets, furniture and curtains), and household products used indoors (e.g., impregnation products and waxes) [47,48,49,50,51,52]. An additional explanation for the more complex PFAS profile in house dust, compared to the other matrices analysed, might be the lower LOQ values achieved for this matrix due to fewer matrix effects during analysis, but this does not explain the higher concentrations found in house dust.

#### 4.1.2. Biotic Matrices

The high presence of PFCAs in matrices such as fruits and vegetables is consistent with previous studies reporting higher accumulation rates for PFCAs than for PFSAs in edible parts of plants [35,53,54]. PFCAs exhibit a lower adsorption affinity for soil particles and plant roots than PFSAs, which makes them more available for plant uptake [55]. The high contribution of short-chain PFAS in small fruits and pods in the present study is comparable to several other studies that investigated similar crops [56,57,58]. The primary route of short-chain PFAS accumulation in above-ground tissues is via passive transport through the xylem vessels, driven by evapotranspiration [59,60], although some studies also suggest active transport routes [61,62]. Shorter chain PFAS are thus more likely to be transported through the xylem vessels, due to their smaller size and higher hydrophilicity [63]. In contrast, the PFAS profile in nuts and in all vegetable categories (except pods) showed a dominance of long chain PFCAs. These findings are consistent with the study of Lasters, Groffen, Eens and Bervoets [19], who measured PFAS in walnuts from private gardens near the same fluorochemical facility, and reported a similar set of detected compounds with concentrations of the same order of magnitude. However, the prevalence of long-chain PFAS in nuts differs from the findings of Groffen, Prinsen, Devos Stoffels, Maas, Vincke, Lasters, Eens and Bervoets [58] who detected only PFBA in *Quercus robur* nuts collected near the same hotspot. Differences in PFAS accumulation among nut, fruit and vegetable species can be due to several factors, including soil contamination profiles, soil characteristics and the plant species-specific physiology [55]. The opportunistic sampling approach across different private gardens may also have contributed to variability in PFAS profiles across species. These findings highlight the importance of the no-regret measures, which advise not relying solely on home-grown vegetables and fruits but ensuring sufficient variety by including store-bought produce. Compared with findings from other international studies near PFAS hotspots, the ∑PFAS concentrations measured in home-grown fruits and vegetables were of the same order of magnitude. Median ∑PFAS concentrations ranged from 1.54 µg/kg in root vegetables to 5.95 µg/kg in pods. Similarly, ∑PFAS levels reported in fruits and vegetables cultivated in residential gardens near a fluorochemical manufacturing facility in North Carolina (USA) ranged from 0.026 to 38 µg/kg [64]. Furthermore, a study conducted in China [65] found that ∑PFAS concentrations in crops within a 10 km radius of a fluorochemical contamination hotspot ranged from 1.36 ng/g to 63.4 ng/g. Finally, the indicative European maximum level for PFOA in food was exceeded at median concentrations in small fruits, tree fruits, leafy vegetables, stem vegetables, fruiting vegetables, and nuts. Similarly, the maximum level established for PFOS was exceeded for leafy vegetables and root vegetables.

The PFAS concentrations in chicken eggs of the present study were similar to those reported in home-produced eggs, collected in 2018, 2019, 2021 and 2022, within the same study area [31]. However, in the study of, PFOS was found to be the major compound and PFBS was rarely detected, whereas in the present study PFBS made up 40% of the profile. Comparable observations were reported for great tit eggs collected in the same area. While Groffen, et al. [66] did not detect PFBS in 2019, a subsequent study by Groffen, et al. [67] identified its presence in these eggs. This suggests that chronic exposure may have contributed to the elevated PFBS levels observed in chicken eggs. Additionally, a shift in production from longer- to shorter-chain PFAS may have further influenced these findings. Furthermore, these differences may reflect local variation in soil PFAS profiles and concentrations across private gardens. When compared with the European maximum levels for PFAS contamination in chicken eggs, 3 of 37 samples exceeded the limit for PFHxS, 22 of 37 surpassed the threshold for PFOA, 1 of 37 exceeded the PFNA limit, and 29 of 37 were above the PFOS limit. For the sum, 29 of 37 egg samples exceeded the established maximum level for the combined concentration of these four compounds. Here, it should be noted that these maximum levels were established for commercial food products. The PFAS detected in the chicken enclosure soil were to some extent in accordance with those found in chicken eggs, but differences in contributions to the ∑PFAS were noticed. Free-ranging laying hens are mainly exposed to persistent pollutants via direct intake of contaminated soil particles [68]. However, the differences in PFAS profiles between chicken enclosure soils and chicken eggs as well as the results of the determinant analyses, indicate that the chickens may also be exposed through additional pathways (e.g., ingestion of soil invertebrates, which were not analysed in this study, commercial feed, food scraps, etc.).

The high dominance of PFOS and PFOA in the serum of the participants reflects the high historical contamination of these compounds within the sampling area. Despite their phase-out, both compounds were persistent in the environment and in humans, which explains their continued prevalence. Additionally, PFOS accumulation in serum might be enhanced since the compound is known to bind with plasma proteins (e.g., albumin) [69]. In a general sample of teenagers recruited across Flanders (Belgium) in 2017–2018 (the Flemish Environment and Health Study; FLEHS-4), only four out of 12 target PFAS were detected in the serum; PFOS, PFOA, PFHxS and PFNA with median concentrations of 2.1 µg/L, 1.0 µg/L, 1.3 µg/L and 0.70 µg/L, for the linear forms, respectively [70]. The present study detected 9 out of 43 analysed PFAS in the serum of the teenagers (i.e., PFOS, PFOA, PFHxS, PFNA, PFDA, PFBA, PFHpS, PFHpA and PFUnDA), but the same four PFAS (PFOS, PFOA, PFHxS and PFNA) dominated the profile, with median concentrations of 2.5 µg/L, 1.1 µg/L, 0.51 µg/L and 0.26 µg/L for the linear forms. Although the median concentrations of these four PFAS were of similar magnitude in both studies, the p95 values for PFOS (linear) was four times higher in the present study (30.0 µg/L) compared to FLEHS-4 (7.30 µg/L). The differences in the amount of PFAS detected between studies may be attributable to the lower LOQs achieved in the present study. Compared to the results from another hotspot in the Veneto Region, Italy, serum PFOS, PFOA, and PFHxS concentrations of the present study were lower [71].

### 4.2. Determinants Associated with PFAS in the Different Environmental Matrices

It should be emphasised that the results of the determinants are based on univariate analyses conducted in relatively small sample sizes. Therefore, some of the observed associations may be driven by unmeasured factors or coincidence, and non-significant associations should not be interpreted as indicating irrelevance. Furthermore, many associations did not remain statistically significant after FDR correction, and the results should therefore be interpreted as indicative rather than confirmatory.

#### 4.2.1. Soil and Compost

PFAS concentrations in soil were associated with several soil characteristics. Positive associations between several long-chain PFAS and TOC were in line with previous studies [72,73,74]. Sorption of PFAS to soil organic matter is likely due to the hydrophobic interactions that occur [72]. Both the proportion of particles <2 µm and pH were significantly associated with PFAS concentrations in the soil. A higher proportion of particles <2 µm increases surface area and can therefore lead to higher PFAS sorption rates in soil. Additionally, a decrease in pH will promote the sorption of anionic PFAS to soil, consistent with the negative associations found in the present study. Finally, dry matter was found to be negatively associated with the PFBS content in the soil of the vegetable garden. PFBS is more hydrophilic than long chain PFAS and therefore more likely to be dissolved in the soil solution compared to being bound to dry matter. Additionally, the composting of weeds seemed to be associated with the PFDoDA concentrations in soil of the vegetable garden, which suggests that PFDoDA is transferred from composted weeds to soil. Only one determinant, i.e., the composting of eggshells, seemed to influence the PFBS concentrations in compost, which is in line with the high presence of PFBS in chicken eggs observed in the present study (although we did not measure PFAS concentrations in eggshells).

#### 4.2.2. Tree Fruits and Fruiting Vegetables

PFAS concentrations in tree fruits and fruiting vegetables were positively associated with composting of grass clippings, weeds and pruning waste, likely reflecting PFAS contamination of these materials in the study area [58,66,75]. It has been documented that contaminated compost is a source of PFAS input to soil [76] and plants [77]. In areas where environmental PFAS concentrations are high, it is advisable not to use your own compost, which is also advised within the local no-regret measures in a 1.5 km radius from the factory.

#### 4.2.3. Chicken Eggs

Several determinants were found for PFAS in chicken eggs. Firstly, larger or polygon-shaped free-range spaces were associated with higher PFTrDA and PFTeDA concentrations in chicken eggs. It is possible that chickens with a larger free-range space will spend more time outdoors increasing their contact with soil particles compared with when they are inside of the chicken coop [78]. A larger foraging space may also stimulate more scratching activity and, consequently higher PFAS concentrations in eggs. Feeding practices were also influential. Hens fed on commercial feed produced eggs with lower PFAS concentrations, suggesting that other feeds (such as scraps of vegetables, invertebrates) contain higher PFAS concentrations. This aligns with the study of Lasters, Groffen, Eens, Coertjens, Gebbink, Hofman and Bervoets [18], where hens that were fed kitchen leftovers had higher egg PFOS and PFOA concentrations, and with the study of Lasters, Van Sundert, Groffen, Buytaert, Eens and Bervoets [30], where the PFBA and PFOS concentrations in vegetables and earthworms, respectively, were significantly positively associated with the egg concentrations. Additionally, feeding weeds to chickens was associated with higher PFAS concentrations in chicken eggs in the present study, likely reflecting PFAS in the weeds or adhering soil. Composting weeds was also associated with higher levels of PFAS in fruits and vegetables, further indicating that weeds may be a significant exposure pathway and should be avoided. Conversely, feeding bread was associated with lower PFAS concentrations, likely due to negligible PFAS concentrations in commercial food products. Some of the variation in PFAS concentrations among egg samples in the present study may be due to the sampling strategy, because eggs were opportunistically sampled and therefore egg samples from different private gardens and different breeds of chickens were collected, which may have already induced some variation in the dataset.

#### 4.2.4. House Dust

Firstly, PFAS in house dust was found to be linked to home renovations. This is consistent with the increase in PFAS levels in house dust during or after home renovations (such as floor waxing) observed in another study [79]. Several studies have reported lower PFAS levels in dust from houses where “healthier” materials were used, compared to conventional materials [51,80]. The present study observed lower PFAS concentrations in houses with mechanical ventilation. Ventilation in combination with particle removal, e.g., by applying filters, can be effective in removing particle-bound chemicals from the indoor environment [81]. Several studies have reported the occurrence of PFAS in dust captured by air conditioning filters [82,83]. Furthermore, cleaning behaviours also seemed to influence the PFAS present in house dust: a higher frequency of brooming was associated with lower 6:2 FTS concentrations, whereas cleaning with water was linked to higher PFPeA concentrations, possibly due to PFPeA in tap water (i.e., up to 14 ng/L in Flanders, 2022) [84]. Cleaning the top of cupboards more frequently also gave rise to lower concentrations indicating that regular cleaning helps remove PFAS-laden dust. Samples from the living room contained higher PFAS concentrations compared to samples from other rooms, which may reflect greater use of consumer products and overall activity in living areas.

## 5. Conclusions

This study identified clear differences in PFAS occurrence across environmental and biological matrices collected near a fluorochemical manufacturing facility in Belgium. PFOS was the most dominant compound in both soil and serum, while PFBA dominated in rainwater, compost, house dust and pods. PFBS was most dominant in fruits and chicken eggs, PFDoDA in rooting vegetables and nuts, and MePFOSAA in fruiting-, stem-, and leafy vegetables. These findings illustrate matrix-specific PFAS accumulation patterns in an area affected by industrial PFAS emissions. Several potential determinants of PFAS concentrations in soil, compost, small and tree fruits, chicken eggs, and house dust were identified and help explain the variability across matrices. However, these associations should be interpreted with caution due to small sample sizes and the univariate nature of the analyses. Future studies would benefit from larger sample sizes and non-opportunistic sampling strategies, enabling separate analysis of individual fruit, vegetable, and nut types. This would allow more robust identification of PFAS determinants and support more confident public health recommendations for residents living near PFAS hotspots.

## Figures and Tables

**Table 1:** Sample sizes per analysed matrix.

Matrix	Sample Size
Soil vegetable garden	62
Soil chicken enclosure	38
Soil greenhouse	10
Compost	36
Chicken eggs	37
Small fruits	29
Tree fruits	33
Leafy vegetables	8
Stem vegetables	17
Fruiting vegetables	22
Pods	6
Rooting vegetables	6
Nuts	7
Rainwater	54
House dust	129
Serum	301

## Data Availability

Data will be made available upon request.

## Supplementary Materials

The following supporting information can be downloaded at: https://www.mdpi.com/article/10.3390/toxics14050360/s1, Text 1: Sampling method. Text 2: PFAS analyses. Text 3: Soil characteristics. Table S1: Determinants of PFAS in soil vegetable garden, compost, tree fruits and small fruits, which were questioned within the questionnaires, with the description of the determinant, the type of variable and the score given based on the answers of the participants. Table S2: Determinants of PFAS in chicken eggs, which were questioned within the questionnaires, with the description of the determinant, the type of variable and the score given based on the answers of the participants. Table S3: Determinants of PFAS in house dust, which were questioned within the questionnaires, with the description of the determinant, the type of variable and the score given based on the answers of the participants. Table S4: Overview of the vegetable food types, classified according to their functional category, which were collected in private gardens within a 5 km radius from a fluorochemical plant site in Antwerp (Belgium). The samples received different pre-treatments, including washing with milli-Q water and/or removal of inedible parts. Table S5: Full names and abbreviations of PFAS measured. Table S6: Full names, abbreviations, MRM transitions (precursor and product ion), internal standard (ISTD) used for quantification, cone voltage (V) and collision energy (eV) for the target PFAS. Table S7: LOQ values per matrix analysed (VG = soil vegetable garden, CE = soil chicken enclosure, GH = soil greenhouse, C = compost, egg = chicken eggs, RW = rainwater, HD = house dust, S = serum), serum concentrations (µg/L), soil and house dust concentrations (µg/kg dry weight), fruits, vegetables, nuts and eggs concentrations (µg/kg fresh weight) and rainwater concentrations (µg/L). Table S8: Median (P25-P75) PFAS concentrations in the different matrices analysed (VG= soil vegetable garden, CE = soil chicken enclosure, GH = soil greenhouse, C = compost, egg = chicken eggs, SF = small fruits, TF = tree fruits, LV = leafy vegetables, SV = stem vegetables, FV = fruiting vegetables, P = pods, RV = rooting vegetables, N = nuts, RW = rainwater, HD = house dust, S = serum), P25-P75 values are only given with N ≥ 12 [29]. Table S9: Detection frequencies of the PFAS analysed in the different matrices analysed expressed in % (VG = soil vegetable garden, CE = soil chicken enclosure, GH = soil greenhouse, C = compost, egg = chicken eggs, SF = small fruits, TF = tree fruits, LV = leafy vegetables, SV = stem vegetables, FV = fruiting vegetables, P = pods, RV = rooting vegetables, N = nuts, RW = rainwater, HD = house dust, S = serum). Table S10: Significant soil characteristics for PFAS concentrations in soil vegetable garden (VG) and soil chicken enclosure (CE). Here, the estimates represent the geometric mean ratio (GMR; for continuous PFAS) or odds ratio (OR; for binary PFAS) for each category compared to the reference category (the category with GMR or OR = 1). Table S11: Significant determinants for PFAS concentrations in soil vegetable garden (VG), soil chicken enclosure (CE), and compost (C). Here, the estimates represent the geometric mean ratio (GMR; for continuous PFAS) or odds ratio (OR; for binary PFAS) for each category compared to the reference category (the category with GMR or OR = 1). Table S12: Significant determinants for PFAS concentrations in tree fruits (TF) and fruiting vegetables (FV). The estimates represent the geometric mean ratio (GMR; for continuous PFAS) or odds ratio (OR; for binary PFAS) for each category compared to the reference category (the category with GMR or OR = 1). Table S13: Significant determinants for PFAS concentrations in chicken eggs. The estimates represent the geometric mean ratio (GMR; for continuous PFAS) or odds ratio (OR; for binary PFAS) for each category compared to the reference category (the category with GMR or OR = 1). Table S14: Significant determinants for PFAS concentrations in house dust. The estimates represent the geometric mean ratio (GMR; for continuous PFAS) or odds ratio (OR; for binary PFAS) for each category compared to the reference category (the category with GMR or OR = 1). Table S15: Significant determinants for PFAS concentrations in house dust. The estimates represent the geometric mean ratio (GMR; for continuous PFAS) or odds ratio (OR; for binary PFAS) for each category compared to the reference category (the category with GMR or OR = 1). Figure S1: Side-by-side comparison of PFAS compositional profiles of chicken eggs obtained using four different substitution methods (MLE substitution, substitution by LOQ/2, substitution by LOQ = 0, and using only samples with concentrations >LOQ), with PFAS that were detected <50% in the samples omitted in all cases. The figures show strong similarity and support the robustness of the MLE substitution approach.

## Abbreviations

The following abbreviations are used in this manuscript:

PFAS	Per- and polyfluoroalkyl substances
EFSA	European Food and Safety Authority
PFOS	Perfluorooctane sulfonic acid
PFOA	Perfluorooctanoic acid
SI	Supplementary Information
OVAM	The Public Waste Agency of Flanders
SPE	Solid-phase extraction
ACN	Acetonitrile
PFHxS	Perfluorohexanesulfonic acid
MePFOSA	N-methylperfluorooctanesulfonamide
EtPFOSA	N-ethylperfluorooctancesulfonamide
MePFOSAA	N-methylperfluorooctanesulfonamido acetic acid
EtPFOSAA	N-ethylperfluorooctanesulfonamido acetic acid
UPLC-MS/MS	Ultra-performance liquid chromatography-coupled tandem mass spectrometry
LOQ	Limit of Quantification
TOC	Total organic carbon
RDC	Research Data Centres of the Federal Statistical Office and the Statistical Offices of the Federal States.
GMR	Geometric Mean Ratio
IQR	Interquartile Range
DW	Dry weight
PFBA	Perfluorobutanoic acid
PFBS	Perfluorobutanesulfonic acid
PFCA	Perfluorocarboxylic acid
PFSA	Perfluorosulfonic acid
PFBSA	Perfluorobutane sulfonamide
PFPeA	Perfluoropentanoic acid
PFHxA	Perfluorohexanoic acid
PFHpA	Perfluoroheptanoic acid
PFDoDA	Perfluorododecanoic acid
PFNA	Perfluorononanoic acid
PFDA	Perfluorodecanoic acid
PFUnDA	Perfluoroundecanoic acid
PFTrDA	Perfluorotridecanoic acid
PFTeDA	Perfluorotetradecanoic acid
PFHpS	Perfluoroheptanesulfonic acid
6:2 FTS	6:2 Fluorotelomer sulfonic acid
SOM	Soil organic matter
HDPE	High density polyethylene
PP	Polypropylene
PCA	Principal component analysis
