# Supplementary material for: From Soil to Serum: Matrix-Specific Per- and Polyfluoroalkyl Substance Accumulation and Potentially Associated Environmental Exposure Determinants in Teenagers Residing near an Industrial Hotspot
Source: Toxics. 2026 Apr 24;14(5):360. doi: 10.3390/toxics14050360 (PMC13211272; doi:10.3390/toxics14050360)
Supplement: Supplementary file 1 [file toxics-14-00360-s001.zip › toxics-4221969-supplementary.pdf]

Supplementary Information

From soil to serum: Matrix-specific PFAS accumulation and potentially associated environmental exposure determinants in teenagers residing near an industrial hotspot

1. **Sampling method**
    a. **Soil samples**

The sampling of soil from the vegetable gardens and chicken runs was carried out in accordance with the guidelines prepared by OVAM. For the soil of the vegetable garden, one pooled sample was taken at each location, using a metal shovel and nitrile gloves. The pooled samples were composed of 10-15 subsamples taken at a depth of 20 cm, well spread throughout the vegetable garden. In the chicken run, one pooled sample was taken at each location, composed of 10-15 subsamples taken at a depth of 10 cm, well spread throughout the chicken run. The subsamples were spread over the entire chicken run, sampling at least three types of locations: the scratching area, the feeding area within the run and the entrance. After each sampling, the shovel was cleaned with tap water and dried with PFAS-free paper. When vegetables or fruits were grown in a greenhouse, a pooled sample was also taken from the soil in the greenhouse. Soil samples were stored in high-density poly-ethylene (HDPE) sealable buckets which were labelled with a unique identification number.

b. **Compost samples**

For compost sampling, 10-15 subsamples were collected at each site scattered over the compost heap, sieved and combined into one mixed sample in a sealable HDPE bucket. Only sievable compost was sampled, excluding decaying food waste and green waste. For a single-vent compost bin, the compost was scooped and sieved into a wheelbarrow. Then, 10-15 subsamples were taken from the wheelbarrow. After each sampling, the scoop, sieve and wheelbarrow were cleaned with tap water and dried with PFAS-free paper.

c. **Rainwater samples**

Rainwater samples were collected in duplicates (2 x 50-ml HDPE bottles per participant). The bottles were filled without air bubbles and were sealed immediately after sampling. When samples were taken from a cistern via a tap, the tap was opened long enough to flush away the dead volume of the tap and pipes (at least 10 L) before taking the samples. When samples were taken from a barrel with a tap, the water from the tap was run for 10 sec. so that the tap was flushed, and then the bottle was filled. When sampling from a barrel without a tap, a scoop sample was taken, avoiding sampling shallow water (min. depth of 5 cm). When samples were taken from a well, a subsample was collected in the same way that participants normally take water from the well (e.g., with a bucket). A scoop sample was then taken from this subsample, avoiding sampling shallow water (min. depth of 5 cm). During transport to the laboratory, the samples were kept in a cooler.

d. **Vegetables, fruits and nuts**

Vegetables, fruits and nuts were sampled during three consecutive months (July–September 2022) and were classified into functional groups as shown in Table 1. Any fruits/vegetables that were present were opportunistically harvested, i.e., whichever was in supply. The samples were transported in HPDE bags under cooled conditions to the laboratory, after which they were washed with milli-Q water. Then, each of the samples received a pre-treatment according to the requirements for human consumption (Table S4). The samples were homogenised with a stainless-steel kitchen mixer (Bosch, type MSM65PER), which was washed with ACN and milli-Q water in between the samples to prevent cross-contamination. The homogenates were stored in the freezer at –20 °C until PFAS extraction and analysis.

e. Chicken eggs

Spread over three weeks, 10 eggs were collected by participants from their own chickens and stored at 4°C until collection. The eggs were immediately transferred to the laboratory in the week of collection and were homogenised using a stainless-steel kitchen mixer (Bosch, type MSM65PER) in a polypropylene (PP) pot. The mixer was washed with ACN and milli-Q water in between the samples to prevent cross-contamination.

f. House dust

Participants were asked to collect house dust using a scraping method. Via mail, participants received a scraper, aluminium foil and a plastic bag. Participants were asked to sweep dust with the scraper, both in the living room and bedroom, on a surface (e.g., on top of a cupboard, a curtained wardrobe, etc.) that had not been cleaned for quite some time (weeks, months or years). The scraper was used to wipe the dust onto the aluminium foil without touching the dust with the hands. The aluminium foil was folded and put into the bag until collection.

g. Blood samples

A peripheral blood sample of 39 mL from all 303 participants of the study was drawn by a physician and spread over multiple primary containers. For PFAS analysis, one of the primary blood samples was centrifuged for 20 minutes at 3500 rpm, and 2.5 mL of serum was stored in a cryo-container at -80°C until further analysis. All samples were registered in the Biobank@VITO specimenbank (Mol, Belgium, ID:BB190064), in accordance with Belgian regulations.

2. PFAS analysis

a. Extraction procedures

A. Soil and compost samples

The samples were prepared in accordance with CMA/5/B and analysed in accordance with CMA/3/D (soil; reflabos.vito.be/2023/CMA_3_D.pdf) and CMA/3/O (compost; https://reflabos.vito.be/2023/CMA_3_O.pdf). The analytical method is summarised as follows. Soil and compost samples were dried overnight at 40 °C. After homogenisation and controlled addition of water to humidify the sample before extraction (amount depending on dry matter content), 5 g of the soil sample was weighed into a PP tube. Then, samples were spiked using 4 ng of a mixture of 10 mass-labelled internal standards ($^{13}C_4$-PFBA, $^{13}C_4$-PFPeA, $^{13}C_2$-PFHxA, $^{13}C_4$-PFOA, $^{13}C_5$-PFNA, $^{13}C_2$-PFDA, $^{13}C_2$-PFUnA, $^{13}C_2$-PFDoA, ^{18}O-PFHxS, $^{13}C_4$-PFOS; Wellington Laboratories, Guelph, ON, Canada), incubated for 15 minutes and subsequently extracted using 25 mL of methanol under shaking. The resulting extract was further purified by solid-phase extraction using activated carbon, which was calculated using the internal standard method. Compost samples were treated differently. After salting out, the organic extract was further cleared using dispersive solid-phase extraction (QuEChERS; PSA and C18). Finally, the purified extract was concentrated under a gentle stream of nitrogen. A 10 µL volume of the processed sample was measured using a liquid chromatograph coupled to a mass spectrometer (UPLC-MS/MS, Waters Acquity Xevo TQ-(X)S with an electrospray interface).

B. Rainwater samples

Full method details are available from reflabos.vito.be/2023/WAC_IV_A_025.pdf. The analytical method is summarised as follows. To 50 mL of a rainwater sample, 4 ng of a mixture of 10 mass-labelled internal standards ($^{13}C_4$-PFBA, $^{13}C_4$-PFPeA, $^{13}C_2$-PFHxA, $^{13}C_4$-PFOA, $^{13}C_5$-PFNA, $^{13}C_2$-PFDA, $^{13}C_2$-PFUnA, $^{13}C_2$-PFDoA, ^{18}O-PFHxS, $^{13}C_4$-PFOS; Wellington Laboratories, Guelph, ON, Canada) was added. The water samples were then extracted using solid-phase extraction of the weak anion-exchange type (WAX). The solid phase was then eluted using methanol and methanol/ammonia. The extract was concentrated under a gentle stream of nitrogen to around 500 µL. The extract was combined with methanol and 10 µL was measured using UPLC-MS/MS (Waters Acquity Xevo TQ-(X)S with an electrospray interface).

C. Vegetables, fruits, nuts and chicken eggs

First, 300 mg of the homogenised sample was placed into a 50 mL polypropylene (PP) tube. Each sample was spiked with 10 ng of a mass-labelled internal standard mixture (MPFAC-MXA, Wellington Laboratories, Guelph, Canada). This is a mixture of seven mass-labelled (^{13}C) perfluoroalkylcarboxylic acids (C_4, C_6, C_8, C_9, C_{10}, C_{11} and C_{12}) and two mass-labelled (^{18}O and ^{13}C) perfluoroalkylsulfonates (C_6 and C_8). Hereafter, 10 mL of acetonitrile (ACN) was added to the samples. After this, samples were vortexed and sonicated in an ultrasonic bath for 3 x 10 min, with vortexing in between. The samples were then extracted overnight on a shaking plate (135 rpm) at room temperature. Afterwards, samples were centrifuged (4°C, 10 min, 2400 rpm, Eppendorf centrifuge 5804R) and the supernatant was transferred into 14 mL PP tubes. Samples were dried until 0.5 mL was reached using a rotational vacuum concentrator (Eppendorf Concentrator 5301). Approximately 25 mg of Envi-Carb graphitized carbon adsorbent was added to a 1.7 mL pp microcentrifuge tube. To the sorbent, 50 µL of glacial acetic acid was added. Samples were then added to the tubes and the 14 mL tubes were rinsed twice using 250 µL of ACN, which was added to the same microcentrifuge tube. Samples were then vortex-mixed for one min and centrifuged for 10 min (10.000 rpm, 4°C, Eppendorf Centrifuge 5415 R). Supernatant was then transferred into a new microcentrifuge tube and dried completely in the rotational vacuum concentrator. The dried eluent was reconstituted with 200 µL of 2% ammonium hydroxide diluted in ACN and vortex-mixed prior to filtration (Powley et al., 2005). Afterwards, samples were filtrated using an ion chromatography Acrodisc 13 mm syringe filter with 0.2 µm Supor polyethersulfone (PES) membrane attached to a PP auto-injector vial.

D. House dust samples

Only house dust samples that contained at least 20 mg were analysed to be able to obtain representative results. Preliminary tests revealed that the sample homogeneity could not be ascertained when a lower sample intake was analysed. The weighed dust samples were placed into Falcon tubes, and 4 ng of a mixture of 10 mass-labelled internal standards ($^{13}C_4$-PFBA, $^{13}C_4$-PFPeA, $^{13}C_2$-PFHxA, $^{13}C_4$-PFOA, $^{13}C_5$-PFNA, $^{13}C_2$-PFDA, $^{13}C_2$-PFUnA, $^{13}C_2$-PFDoA, ^{18}O-PFHxS, $^{13}C_4$-PFOS; Wellington Laboratories, Guelph, ON, Canada) was added to the samples. Then, 4 mL of methanol was added and samples were sonicated for 1 hour and vortex-mixed 4 times during the 1-hour extraction. After extraction, samples were centrifuged, and the supernatant was concentrated under a gentle stream of nitrogen and back-extracted using solid-phase extraction of the ENVICARB-type. The SPE cartridge was eluted using acetonitrile. The cleaned extract was further concentrated, combined with methanol, and 10 µL of this extract was measured using UPLC-MS/MS (Waters Acquity Xevo TQ-(X)S with an electrospray interface).

E. Serum samples

The samples were stored at -20 °C prior to analysis. After thawing and homogenisation, 4 ng of a mixture of 10 mass-labelled internal standards was added to the 500 µL serum ($^{13}C_4$-PFBA, $^{13}C_4$-PFPeA, $^{13}C_2$-PFHxA, $^{13}C_4$-PFOA, $^{13}C_5$-PFNA, $^{13}C_2$-PFDA, $^{13}C_2$-PFUnA, $^{13}C_2$-PFDoA, ^{18}O-PFHxS, $^{13}C_4$-PFOS; Wellington Laboratories, Guelph, ON, Canada). Methanol was then added for protein precipitation, and the precipitate was removed by centrifugation. Of the processed sample, 10 µL was adjusted to the starting gradient and was measured using UPLC-MS/MS (Waters Acquity Xevo TQ-(X)S with an electrospray interface).

b. UPLC-MS/MS analysis

A. Soil, compost, rainwater, house dust and serum samples

All samples were measured using ultra-performance liquid chromatography coupled with tandem electrospray (negative) mass spectrometry (UPLC-ESI-MS/MS). Analysis was performed using water- and MeOH-based gradient elution at a flow rate of 300 µL/min and a column temperature of 40 °C. All measurements were done in Multiple Reaction Monitoring (MRM) mode. The method used has a previously validated measurement range of 0.2 - 30 µg/L PFAS in serum. Each measurement series consisted of 20 samples and was accompanied by the necessary quality control measurements: control standards (measurement standards for calibration and integration standards for evaluation of branched isomers) and a control serum sample. The method quality of all analyses except house dust is ensured through a BELAC ISO 17025 accreditation (BELTEST-045).

B. Vegetables, fruits, nuts and eggs

All samples were investigated for 21 PFAS using ultra-performance liquid chromatography coupled with tandem electrospray (negative) mass spectrometry (UPLC-ESI-MS/MS) (Table 2). An ACQUITY BEH C18 column (2.1 x 50 mm; 1.7 µm) was used to separate the analytes. To retain any PFAS contamination originating from the system, an ACQUITY BEH C18 pre-column (2.1 x 30 mm; 1.7 µm) was placed between the solvent mixer and the injector. The mobile phase solvents used were 0.1% formic acid in water (A) and 0.1% formic acid in ACN (B), using a flow rate of 450 µL/min and an injection volume of 6 µL (partial loop). The gradient started at 65% A, decreased to 0% A in 3.4 min and returned to 65% A at 4.7 min. PFAS were identified and quantified using multiple reaction monitoring (MRM) of 2 diagnostic transitions per target analyte as validated by Groffen et al. (2021).

3. Soil characteristics

From the soil samples, moisture content, clay content, total organic carbon (TOC) content and pH were determined. Moisture content was determined by drying the samples in an oven at 105°C until the weight remained stable, and dry matter was then expressed in percentages (ASTM, 2019). The clay content was determined by performing a texture analysis in which the grain size fractions of the mineral soil are separated and determined (sand, loam and clay). The analysis was carried out on the fine soil (<2 mm), after separation of the coarse elements. To obtain a good dispersion of the clay fraction, all cementing materials such as organic material, $CaCO_3$, oxides and dissolved sands were removed. The fine fractions (loam and clay) were separated from the sand by wet sieving on a 50 or 63 µm sieve. The clay content (<63 µm) was then determined using a Robinson–Köhn pipette after dispersion of the colloidal fraction with a dispersing agent (ISO, 2020). The total organic carbon content was determined by using the direct method, in which the carbonates present in the sample are removed by treating the sample with hydrochloric acid (NBN, 2022). The amount of CO_2 that was released on the subsequent combustion was then measured and is a direct measure of the TOC content. At last, pH was determined

by means of a potential measurement using two calibrated electrodes (ISO, 2021). More details can be found on the procedure portal of the Flemish Reference Laboratory for the Environment (emis.vito.be).

Tables:

Table S1: Determinants of PFAS in soil vegetable garden, compost, tree fruits and fruiting vegetables, which were assessed within the questionnaires, with the description of the determinant, the type of variable and the score given based on the answers of the participants.

Description of determinant	Type of variable	Matrix tested	Score
Lutum (clay) content	Numerical	Soil chicken enclosure, soil vegetable garden	%
Total organic carbon content	Numerical	Soil chicken enclosure, soil vegetable garden	%
Dry matter	Numerical	Soil chicken enclosure, soil vegetable garden	%
pH	Numerical	Soil chicken enclosure, soil vegetable garden	Numerical
Type of water used to water vegetable garden	Categorical	Soil vegetable garden	1 = water from rain barrel/cistern<br>2 = ground water<br>3 = tap water<br>4 = other
Type of watering device used to water vegetable garden	Categorical	Soil vegetable garden, tree fruits, fruiting vegetables	0 = garden hose<br>1 = plastic watering can
Pruning waste thrown on compost?	Categorical	Soil vegetable garden, compost, tree fruits	0 = never/occasionally<br>1 = regularly/always
Weeds thrown on compost?	Categorical	Soil vegetable garden, compost, tree fruits, fruiting vegetables	0 = never/occasionally<br>1 = regularly/always
Grass thrown on compost	Categorical	Soil vegetable garden, compost, tree fruits, fruiting vegetables	0 = never/occasionally<br>1 = regularly/always
Vegetable or fruit scraps from own produce thrown on compost?	Categorical	Soil vegetable garden, compost, tree fruits, fruiting vegetables	0 = never/occasionally<br>1 = regularly/always
Vegetable or fruit scraps not from own produce thrown on compost?	Categorical	Soil vegetable garden, compost, tree fruits, fruiting vegetables	0 = never/occasionally<br>1 = regularly/always

Egg shells thrown on compost?	Categorical	Soil vegetable garden, compost, tree fruits	0 = never/occasionally<br>1 = regularly/always

Table S2: Determinants of PFAS in chicken eggs, which were assessed within the questionnaires, with the description of the determinant, the type of variable and the score given based on the answers of the participants.

Description of determinant	Type of variable	Score
What is the surface area of the chicken enclosure?	Categorical	0 = ≤ 20 m²<br>1 = > 20 m²
What is the shape of the chicken enclosure?	Categorical	0 = square/rectangular<br>1 = polygonal
What is the age of the chicken enclosure?	Categorical	1 = < 5 years old<br>2 = between 5 and 10 years old<br>3 = between 10 and 20 years old<br>4 = > 20 years
How often do you feed the chickens commercial chicken feed?	Categorical	0 = regularly<br>1 = always
Do the chickens eat grass clippings?	Categorical	0 = never/occasionally<br>1 = regularly/always
Do the chickens eat weeds?	Categorical	0 = never/occasionally<br>1 = regularly/always
Do the chickens eat bread?	Categorical	0 = never/occasionally<br>1 = regularly/always
Do the chickens eat vegetable/fruit scraps from own produce?	Categorical	0 = never/occasionally<br>1 = regularly/always
Do the chickens eat whole vegetables/fruits from own produce?	Categorical	0 = never/occasionally<br>1 = regularly/always
Do the chickens eat food scraps?	Categorical	0 = never/occasionally<br>1 = regularly/always
Do the chickens have access to the compost heap?	Categorical	0 = no<br>1 = yes

Table S3: Determinants of PFAS in house dust, which were assessed within the questionnaires, with the description of the determinant, the type of variable and the score given based on the answers of the participants.

Description of determinant	Type of variable	Score
In which room was the dust collected using the scraper	Categorical	1 = living room<br>2 = bedroom<br>3 = both<br>4 = other
Was the dust scraped from the top of a cabinet?	Categorical	0 = no<br>1 = yes
When was the last cleaning of that surface?	Categorical	1 = one year ago or longer<br>2 = more than 3 months ago<br>3 = less than 3 months ago
Were there renovations during sampling?	Catgorical	0 = no<br>1 = yes
Was the living room mechanically ventilated?	Catgorical	0 = no<br>1 = yes
Method of ventilation in living room	Catgorical	1 = manually<br>2 = grills<br>3 = mechanically
Was the bedroom mechanically ventilated?	Catgorical	0 = no<br>1 = yes
Method of ventilation in bedroom	Catgorical	1 = manually<br>2 = grills<br>3 = mechanically
Mechanical ventilation in living room or bedroom?	Catgorical	0 = no<br>1 = yes
Frequency of ventilating living room	Categorical	1 = never<br>2 = daily (short)<br>3= daily (long)
Frequency of ventilating bedroom	Categorical	1 = never<br>2 = daily (short)<br>3= daily (long)
Frequency of vacuuming living room	Categorical	0 = weekly or less<br>1 = multiple times a week
Frequency of sweeping living room	Categorical	0 = weekly or less<br>1 = multiple times a week
Frequency of dusting living room	Categorical	0 = weekly or less<br>1 = multiple times a week
Frequency of cleaning living room with water	Categorical	0 = weekly or less<br>1 = multiple times a week
Frequency of vacuuming bedroom	Categorical	0 = weekly or less<br>1 = multiple times a week
Frequency of sweeping bedroom	Categorical	0 = weekly or less<br>1 = multiple times a week
Frequency of dusting bedroom	Categorical	0 = weekly or less<br>1 = multiple times a week

Frequency of cleaning bedroom with water	Categorical	0 = weekly or less<br>1 = multiple times a week

Table S4: Overview of the vegetable food types, classified according to their functional category, which were collected in private gardens within a 5 km radius from a fluorochemical plant site in Antwerp (Belgium). The samples received different pre-treatments, including washing with milli-Q water and/or removal of inedible parts.

Category	Type	Pre-treatment
Nut (*N* = 7)	walnut	Removal of shell
	hazelnut	Removal of shell
Tree fruit (*N* = 33)	apple	Rinsing with MilliQ and removal of seeds
	pear	Rinsing with MilliQ and removal of seeds
	plum	Rinsing with MilliQ and removing seeds
	fig	Removing peel
Small fruit (*N* = 29)	grape	Rinsing with MilliQ
	blackberry	Rinsing with MilliQ
	blueberry	Rinsing with MilliQ
	strawberry	Rinsing with MilliQ
	redcurrant	Rinsing with MilliQ
	kiwiberry	Rinsing with MilliQ
	passion fruit	Removing peel
Root vegetable (*N* = 6)	carrot	Rinsing with MilliQ and removal of top
	beetroot	Rinsing with MilliQ and removal of peel
Fruit vegetable (*N* = 22)	paprika	Rinsing with MilliQ and removal of seeds and top
	courgette	Rinsing with MilliQ and removal of top
	cucumber	Rinsing with MilliQ and removal of top
	pumpkin	Rinsing with MilliQ and removal of seeds and top
	tomato	Rinsing with MilliQ and removal of green top
	pickle	Rinsing with MilliQ and removal of top
Stem vegetable (*N* = 17)	rhubarb	Rinsing with MilliQ and removal of peel
	celery	Rinsing with MilliQ
	leak	Rinsing with MilliQ
Leafy vegetable (*N* = 8)	lettuce	Rinsing with MilliQ
	spinach	Rinsing with MilliQ
	warmos	Rinsing with MilliQ
Pod (*N* = 6)	bean	Rinsing with MilliQ and removal of top
	legume	Rinsing with MilliQ and removal from the pod

Table S5: Full names and abbreviations of PFAS measured

Full name	Abbreviation	Final selection
Perfluorobutanoic acid	PFBA	Yes
Perfluoropentanoic acid	PFPeA	Yes
Perfluorohexanoic acid	PFHxA	Yes
Perfluoroheptanoic acid	PFHpA	Yes
Perfluorooctanoic acid	PFOA	Yes
Perfluorooctanoic acid (linear + branched)	PFOA	Yes
Perfluorononanoic acid	PFNA	Yes
Perfluorododecanoic acid	PFDA	Yes
Perfluoroundecanoic acid	PFUnDA	Yes
Perfluorododecanoic acid	PFDoDA	Yes
Perfluorotridecanoic acid	PFTrDA	Yes
Perfluorotetradecanoic acid	PFTeDA	Yes
Perfluorohexadecanoic acid	PFHxDA	Yes
Perfluorooctadecanoic acid	PFODA	No
Perfluorobutanesulfonic acid	PFBS	Yes
Perfluoropentanesulfonic acid	PFPeS	No
Perfluorohexanesulfonic acid	PFHxS	Yes
Perfluorohexanesulfonic acid (linear + branched)	PFHxS	Yes
Perfluoroheptanesulfonic acid	PFHpS	Yes
Perfluorooctanesulfonic acid	PFOS	Yes
Perfluorooctanesulfonic acid (linear + branched)	PFOS	Yes
Perfluorononanesulfonic acid	PFNS	No
Perfluorododecanesulfonic acid	PFDS	No
Perfluorododecanesulfonic acid	PFDoDS	No
Perfluorobutanesulfonamide	PFBSA	Yes
Perfluorohexanesulfonamide	PFHxSA	No
Perfluorooctanesulfonamide	PFOSA	No
N-methylperfluorooctane sulfonamide	MePFOSA	No
N-methylperfluorooctane sulfonamide (linear + branched)	MePFOSA	No
N-ethylperfluorooctanesulfonamide	EtPFOSA	No
N-ethylperfluorooctanesulfonamide (linear + branched)	EtPFOSA	No

Perfluorooctanesulfonamidoacetic acid	PFOSAA	No
N-methylperfluorooctane sulfonamidoacetic acid	MePFOSAA	Yes

Table S5 continued: Full names and abbreviations of PFAS measured

Full name	Abbreviation	Final selection
N-methylperfluorooctane sulfonamidoacetic acid (linear + branched)	MePFOSAA	Yes
N-ethylperfluorooctane sulfonamidoacetic acid	EtPFOSAA	Yes
N-ethylperfluorooctane sulfonamidoacetic acid (linear + branched)	EtPFOSAA	Yes
4:2 fluorotelomer sulfonic acid	4:2 FTS	No
6:2 fluorotelomer sulfonic acid	6:2 FTS	Yes
8:2 fluorotelomer sulfonic acid	8:2 FTS	No
10:2 fluorotelomer sulfonic acid	10:2 FTS	No
6:2 fluorotelomer phosphate diester	6:2 diPAP	Yes
6:2/8:2 fluorotelomer phosphate diester	6:2/8:2diPAP	No
8:2 fluorotelomer phosphate diester	8:2 diPAP	No
Perfluoro-2-methyl-3-oxahexanoic acid	HFPO-DA/GenX	No
3H-perfluoro-4,8-dioxanonanoic acid	NaDONA	No
Perfluoroethylcyclohexane sulfonate	PFECHS	No
Perfluoro(2-((6-chlorohexyl)oxy)ethanesulfonic acid	9Cl-PF3ONS	No
11-chloroperfluoro-3-oxadecanesulfonic acid	11Cl-PF3OUdS	No

Table S6: Full names, abbreviations, MRM transitions (precursor and product ion), internal standard (ISTD) used for quantification, cone voltage (V) and collision energy (eV) for the target PFAS

**Analyte**		**ISTD used for quantification**	**Precursor ion (*m/z*)**	**Product ion (*m/z*)**		**Collision Energy (eV)**		**Cone Voltage (V)**	
**Full Name**	**Abbreviation**			**Diagnostic product ion 1**	**Diagnostic product ion 2**	**Diagnostic product ion 1**	**Diagnostic product ion 2**	**Diagnostic product ion 1**	**Diagnostic product ion 2**
Perfluorobutanoic acid	PFBA	$^{13}C_4$-PFBA	213	169	169	19	50	19	-
Perfluoropentanoic acid	PFPeA	$^{13}C_4$-PFBA	263	219	219	10	45	15	-
Perfluorohexanoic acid	PFHxA	[1,2-$^{13}C_2$]PFHxA	313	269	119	21	65	19	-
Perfluoroheptanoic acid	PFHpA	[1,2-$^{13}C_2$]PFHxA	363	319	169	40	30	24	-
Perfluorooctanoic acid	PFOA	[1,2,3,4-$^{13}C_2$]PFOA	413	369	169	13	60	22	-
Perfluorononanoic acid	PFNA	[1,2,3,4,5-$^{13}C_2$]PFNA	463	419	169	17	20	28	-
Perfluorododecanoic acid	PFDA	[1,2-$^{13}C_2$]PFDA	513	469	219	29	29	25	-
Perfluoroundecanoic acid	PFUnDA	[1,2-$^{13}C_2$]PFUnDA	563	519	169	30	35	18	-
Perfluorododecanoic acid	PFDoDA	[1,2-$^{13}C_2$]PFDoDA	613	569	319	30	30	22	-
Perfluorotridecanoic acid	PFTrDA	[1,2-$^{13}C_2$]PFDoDA	663	619	319	21	30	26	-
Perfluorotetradecanoic acid	PFTeDA	[1,2-$^{13}C_2$]PFDoDA	713	669	169	21	21	28	-

Perfluorohexadecanoic acid	PFHxDA	[1,2-$^{13}C_2$]PFUnDA	813	769	719	15	15	10	
Perfluorobutanesulfonic acid	PFBS	$^{18}O_2$-PFHxS	299	80	99	65	45	40	-
Perfluorohexanesulfonic acid	PFHxS	$^{18}O_2$-PFHxS	399	80	99	30	60	22	-
Perfluoroheptanesulfonic acid	PFHpS	[1,2,3,4-$^{13}C_2$]PFOA	449	80	98.5	47	45	40	-
Perfluorooctanesulfonic acid	PFOS	[1,2,3,4-$^{13}C_4$]PFOS	499	80	99	58	58	60	-
Perfluorobutanesulfonamide	PFBSA	$^{13}C_4$-PFBA	298	80	219	40	34	38	27
N-methylperfluorooctane sulfonamidoacetic acid	MePFOSAA	[1,2-$^{13}C_2$]PFHxA	570	483	419	29	36	25	
N-ethylperfluorooctane sulfonamidoacetic acid	EtPFOSAA	[1,2-$^{13}C_2$]PFHxA	584	526	419	36	37	28	
6:2 fluorotelomer sulfonic acid	6:2 FTS	[1,2,3,4-$^{13}C_4$]PFOS	427	407	80	25	33	20	-
6:2 fluorotelomer phosphate diester	6:2 diPAP	[1,2,3,4-$^{13}C_2$]PFOA	789	442.7	96.7	30	37	31	-

Table S7: Mean procedural blank values measured during the analyses of fruits, vegetables and nuts, and chicken eggs (µg/kg)

Compound	Mean PB values (fruits, vegetables and nuts (µg/kg))	Mean PB values (chicken eggs (µg/kg))
PFBA	0	0
PFPeA	0	0.073
PFHxA	0.001	0.925
PFHpA	0.003	0.152
PFOA	0.002	0.775
PFNA	0.001	0.097
PFDA	0.001	1.30
PFUnDA	0.004	0.577
PFDoDA	0.003	1.23
PFTrDA	0	0.877
PFTeDA	0.001	0.134
PFHxDA	0	0
PFBS	0.002	1.01
PFHxS	0	0
PFHpS	0	0
PFOS	0	0.600
PFBSA	0	0
MePFOSAA	0	0
EtPFOSAA	0	0
6:2 FTS	0	0.012
6:2 diPAP	0	0

Table S8: LOQ values per matrix analysed (VG= soil vegetable garden, CE = soil chicken enclosure, GH = soil greenhouse, C = compost, egg = chicken eggs, RW = rainwater, HD = house dust, S = serum), serum concentrations (µg/L), soil and house dust concentrations (µg/kg dry weight), fruits, vegetables, nuts and egg concentrations (µg/kg fresh weight) and rainwater concentrations (µg/L).

LOQ values	VG (µg/kg dw)	CE (µg/kg dw)	GH (µg/kg dw)	C (µg/kg dw)	Egg (µg/kg fw)	Vegetables (µg/kg fw)	Fruits (µg/kg fw)	Nuts (µg/kg fw)	RW (µg/L)	HD (µg/kg dw)	S (µg/L)
**PFBA**	[0.04;0.1]	[0.04;0.1]	[0.04;0.1]	[0.04;0.1]	0.11	0.22	0.22	0.23	[0.0009;0.002]	[1.0;7.0]	0.1
**PFPeA**	[0.04;0.4]	[0.04;0.4]	[0.04;0.4]	[0.04;0.4]	0.14	0.10	0.08	0.09	[0.0009;0.001]	[0.4;7.0]	0.1
**PFHxA**	[0.04;0.4]	[0.04;0.4]	[0.04;0.4]	[0.04;0.4]	0.10	0.23	0.12	0.13	[0.001;0.002]	[0.5;2.0]	0.1
**PFHpA**	[0.05;1.00]	[0.05;1.00]	[0.05;1.00]	[0.05;1.00]	0.06	0.24	0.21	0.19	[0.0009;0.001]	[0.2;4.0]	[0.1;0.3]
**PFOA**	[0.2;0.4]	[0.2;0.4]	[0.2;0.4]	[0.2;0.4]	0.15	0.13	0.11	0.11	[0.0008;0.001]	[2.0;10]	0.1
**$PFOA_{linear+branched}$**	[0.2;0.4]	[0.2;0.4]	[0.2;0.4]	[0.2;0.4]	0.15	0.13	0.11	0.11	0.0008	[3.0;20]	0.1
**PFNA**	[0.05;0.4]	[0.05;0.4]	[0.05;0.4]	[0.05;0.4]	0.15	0.04	0.02	0.03	[0.0009;0.001]	[0.4;2.0]	0.1
**PFDA**	[0.05;0.4]	[0.05;0.4]	[0.05;0.4]	[0.05;0.4]	0.20	0.07	0.09	0.03	[0.0009;0.001]	[1.0;2.0]	0.1
**PFUnDA**	[0.05;0.4]	[0.05;0.4]	[0.05;0.4]	[0.05;0.4]	0.14	0.06	0.04	0.09	[0.0009:0.002]	[0.2;6.0]	0.1
**PFDoDA**	[0.05;0.4]	[0.05;0.4]	[0.05;0.4]	[0.05;0.4]	0.49	0.26	0.22	0.06	[0.0009;0.003]	[0.2;2.0]	0.1
**PFTrDA**	[0.05;0.4]	[0.05;0.4]	[0.05;0.4]	[0.05;0.4]	0.34	0.08	0.08	0.24	[0.0009;0.003]	[0.2;2.0]	[0.1;0.16]
**PFTeDA**	[0.05;0.4]	[0.05;0.4]	[0.05;0.4]	[0.05;0.4]	0.55	0.03	0.28	0.22	[0.0009;0.004]	[0.1;0.3]	0.1
**PFHxDA**	[0.05;0.4]	[0.05;0.4]	[0.05;0.4]	[0.05;0.4]	0.04	0.02	0.04	0.02	[0.0009;0.003]	[0.2;6.0]	0.1
**PFBS**	[0.04;0.05]	[0.04;0.05]	[0.04;0.05]	[0.04;0.05]	0.38	0.62	0.66	0.16	[0.0009;0.001]	[0.07;0.5]	0.1

**PFHxS**	[0.04;0.4]	[0.04;0.4]	[0.04;0.4]	[0.04;0.4]	0.48	0.91	0.62	0.49	[0.0009;0.001]	[0.03;0.2]	0.1
**PFHxS**$_{linear+branched}$	[0.04;0.4]	[0.04;0.4]	[0.04;0.4]	[0.04;0.4]	0.48	0.91	0.62	0.49	[0.0009;0.001]	[0.03;0.2]	0.1
**PFHpS**	[0.04;0.4]	[0.04;0.4]	[0.04;0.4]	[0.04;0.4]	0.51	0.04	0.30	0.32	[0.0009;0.001]	[0.02;0.3]	[0.1;0.2]
**PFOS**	0.2	0.2	0.2	0.2	0.10	0.02	0.02	0.02	[0.002;0.008]	[4.0;20]	0.1
**PFOS**$_{linear+branched}$	0.5	0.5	0.5	0.5	0.10	0.02	0.02	0.02	[0.002;0.01]	[6.0;20]	0.1
**PFBSA**	[0.05;0.4]	[0.05;0.4]	[0.05;0.4]	[0.05;0.4]	0.20	0.13	0.10	0.16	[0.0009;0.004]	[0.05;0.9]	0.1
**MePFOSAA**	[0.04;0.2]	[0.04;0.2]	[0.04;0.2]	[0.04;0.2]	0.77	0.37	0.92	0.68	[0.0009;0.001]	[0.1;0.9]	0.1
**MePFOSAA**$_{linear+branched}$	[0.04;0.2]	[0.04;0.2]	[0.04;0.2]	[0.04;0.2]	0.77	0.37	0.92	0.68	[0.0009;0.001]	[0.2;0.9]	0.1
**EtPFOSAA**	[0.05;0.2]	[0.05;0.2]	[0.05;0.2]	[0.05;0.2]	0.35	0.17	0.42	0.27	[0.0009;0.001]	[0.1;1.0]	0.1
**EtPFOSAA**$_{linear+branched}$	[0.05;0.2]	[0.05;0.2]	[0.05;0.2]	[0.05;0.2]	0.35	0.17	0.42	0.27	[0.0009;0.001]	[0.1;1.0]	0.1
**6:2 FTS**	[0.04;0.4]	[0.04;0.4]	[0.04;0.4]	[0.04;0.4]	0.34	0.25	0.18	0.12	[0.0006;0.003]	[0.3;2.0]	0.1
**6:2 diPAP**	[0;30.0]	[0;30.0]	[0;30.0]	[0;30.0]	1.00	0.52	1.3	0.85	[0.0009;0.004]	-	0.1

Table S9: Median (P25-P75) PFAS concentrations in the different matrices analysed (VG= soil vegetable garden, CE = soil chicken enclosure, GH = soil greenhouse, C = compost, egg = chicken eggs, SF = small fruits, TF = tree fruits, LV = leafy vegetables, SV = stem vegetables, FV = fruiting vegetables, P = pods, RV = rooting vegetables, N = nuts, RW = rainwater, HD = house dust, S = serum). P25-P75 values are only given with N ≥ 12 (RDC, 2022).

Median concentrations	VG (n = 62)	CE (n = 38)	GH (n = 10)	C (n = 36)	Egg (n = 37)	SF (n = 29)	TF (n = 33)	LV (n = 8)	SV (n = 17)	FV (n = 22)	P (n = 6)	RV (n = 6)	N (n = 7)	RW (n = 54)	HD (n = 129)	S (n = 301)
**PFBA**	0.31 (0.18 – 0.61)	0.23 (<LOQ – 0.61)	<LOQ	1.30 (0.72 – 2.30)	<LOQ	<LOQ	<LOQ (<LOQ – 0.77)	<LOQ	<LOQ	<LOQ	2.65	<LOQ	<LOQ	0.005 (<LOQ – 0.01)	14.0 (5.30 – 30.0)	0.15 (<LOQ – 0.19)
**PFPeA**	0.07 (<LOQ – 0.13)	<LOQ (<LOQ – 0.07)	0.11	<LOQ (<LOQ – 0.34)	<LOQ	0.13 (<LOQ – 0.22)	<LOQ	<LOQ	<LOQ	<LOQ	<LOQ	<LOQ	0.40	<LOQ (<LOQ – 0.003)	1.50 (0.84 – 2.30)	<LOQ
**PFHxA**	0.07 (0.05 – 0.11)	0.06 (<LOQ – 0.09)	0.14	<LOQ (<LOQ – 0.22)	0.30 (<LOQ – 0.50)	<LOQ (<LOQ – 0.53)	0.34 (<LOQ – 0.50)	<LOQ	0.42 (<LOQ – 0.59)	<LOQ (<LOQ – 0.40)	<LOQ	0.29	<LOQ	0.004 (0.002 – 0.009)	5.20 (<LOQ – 14.0)	<LOQ
**PFHpA**	0.06 (<LOQ – 0.08)	<LOQ (<LOQ – 0.08)	0.08	<LOQ	<LOQ	<LOQ	<LOQ	<LOQ	<LOQ	<LOQ	1.28	<LOQ	<LOQ	0.002 (0.001 – 0.003)	2.50 (1.00 – 4.20)	<LOQ
**PFOA**	0.25 (0.20 – 0.39)	0.24 (0.20 – 0.33)	0.29	0.43 (0.27 – 0.62)	0.36 (0.18 – 0.65)	0.13 (<LOQ – 0.24)	0.11 (<LOQ – 0.17)	0.21	0.13 (<LOQ – 0.17)	0.13 (<LOQ – 0.20)	<LOQ	<LOQ	0.15	0.005 (0.003 – 0.008)	11.0 (5.70 – 17.0)	1.10 (0.87 – 1.40)
**$PFOA_{linear+branched}$**	0.27 (0.20 – 0.40)	0.24 (0.20 – 0.34)	0.31	0.44 (0.30 – 0.66)	0.36 (0.18 – 0.65)	0.13 (<LOQ – 0.24)	0.11 (<LOQ – 0.17)	0.21	0.13 (<LOQ – 0.17)	0.13 (<LOQ – 0.20)	<LOQ	<LOQ	0.15	0.005 (0.003 – 0.009)	12.0 (6.20 – 19.0)	1.10 (0.92 – 1.50)
**PFNA**	0.09 (0.06 – 0.12)	0.07 (<LOQ – 0.10)	0.07	<LOQ	0.16 (<LOQ – 0.24)	<LOQ	<LOQ	<LOQ	<LOQ	<LOQ	<LOQ	<LOQ	<LOQ	0.001 (<LOQ – 0.002)	3.00 (1.30 – 4.80)	0.26 (0.19 – 0.20)

**PFDA**	0.11 (0.07 – 0.15)	0.08 (0.06 – 0.14)	0.11	<LOQ	0.83 (0.61 – 0.98)	0.54 (0.37 – 0.62)	0.44 (0.37 – 0.54)	0.50	0.50 (0.40 – 0.56)	0.54 (0.42 – 0.75)	0.50	0.40	0.60	0.001 (<LOQ – 0.002)	3.65 (1.95 – 8.45)	0.14 (<LOQ – 0.20)
**PFUnDA**	<LOQ (<LOQ – 0.07)	<LOQ (<LOQ – 0.06)	<LOQ	<LOQ	0.15 (<LOQ – 0.35)	<LOQ (<LOQ – 0.14)	0.12 (<LOQ – 0.14)	<LOQ	<LOQ (<LOQ – 0.20)	<LOQ (<LOQ – 0.17)	<LOQ	0.12	0.20	<LOQ	1.50 (<LOQ – 3.10)	<LOQ
**PFDoDA**	<LOQ (<LOQ – 0.07)	<LOQ (<LOQ – 0.05)	<LOQ	<LOQ	1.88 (1.17 – 2.58)	0.58 (<LOQ – 0.81)	0.55 (0.22 – 0.71)	0.78	0.56 (0.26 – 0.74)	0.59 (<LOQ – 0.88)	<LOQ	0.58	1.01	<LOQ	<LOQ (<LOQ – 5.00)	<LOQ
**PFTrDA**	<LOQ	<LOQ	<LOQ	<LOQ	0.61 (<LOQ – 1.21)	<LOQ (<LOQ – 0.23)	<LOQ (<LOQ – 0.12)	<LOQ	<LOQ (<LOQ – 0.16)	<LOQ	<LOQ	<LOQ	<LOQ	<LOQ	0.50 (<LOQ – 1.20)	<LOQ
**PFTeDA**	<LOQ	<LOQ	<LOQ	<LOQ	1.36 (<LOQ – 2.39)	<LOQ	<LOQ	<LOQ	<LOQ	<LOQ	<LOQ	<LOQ	<LOQ	<LOQ	1.10 (0.53 – 2.10)	<LOQ
**PFHxDA**	<LOQ	<LOQ	<LOQ	<LOQ	<LOQ	0.16 (<LOQ – 0.25)	0.16 (0.10 – 0.20)	0.23	0.19 (0.16 – 0.21)	0.15 (0.04 – 0.21)	0.20	0.13	0.18	<LOQ	0.54 (0.22 – 1.40)	<LOQ
**PFBS**	0.12 (0.07 – 0.24)	0.14 (0.07 – 0.28)	0.23	1.40 (0.62 – 2.35)	3.34 (1.74 – 7.41)	2.99 (<LOQ – 12.79)	0.85 (<LOQ – 5.58)	<LOQ	<LOQ (<LOQ – 4.37)	<LOQ (<LOQ – 4.14)	<LOQ	<LOQ	<LOQ	0.002 (<LOQ – 0.006)	1.95 (1.10 – 4.00)	<LOQ
**PFHxS**	<LOQ	<LOQ (<LOQ – 0.06)	<LOQ	<LOQ	<LOQ	<LOQ	<LOQ	<LOQ	<LOQ	<LOQ	<LOQ	<LOQ	<LOQ	<LOQ	0.54 (0.26 – 1.10)	0.51 (0.36 – 0.83)
**$PFHxS_{linear+branched}$**	<LOQ (<LOQ – 0.06)	<LOQ (<LOQ – 0.07)	0.05	<LOQ	<LOQ	<LOQ	<LOQ	<LOQ	<LOQ	<LOQ	<LOQ	<LOQ	<LOQ	<LOQ	0.69 (0.33 – 1.30)	0.54 (0.38 – 0.87)

**PFHpS**	<LOQ	<LOQ	<LOQ	<LOQ	<LOQ	<LOQ	<LOQ	<LOQ	<LOQ	<LOQ	<LOQ	<LOQ	<LOQ	<LOQ	0.10 (<LOQ – 0.21)	<LOQ
**PFOS**	2.30 (1.40 – 3.90)	2.15 (1.50 – 2.90)	1.65	1.75 (1.25 – 2.45)	3.09 (1.59 – 4.95)	<LOQ	<LOQ	0.02	<LOQ (<LOQ – 0.03)	<LOQ	<LOQ	0.02	<LOQ	0.004 (<LOQ – 0.051)	12.0 (7.40 – 22.0)	2.50 (1.40 – 5.50)
**PFOS$_{linear+branched}$**	2.75 (1.70 – 4.40)	2.60 (1.80 – 3.40)	2.30	2.25 (1.60 – 3.20)	3.28 (1.74 – 6.56)	<LOQ	<LOQ	0.03	<LOQ (<LOQ – 0.03)	<LOQ	<LOQ	0.02	<LOQ	0.005 (<LOQ – 0.008)	19.0 (11.0 – 33.0)	7.30 (4.80 – 13.00)
**PFBSA**	0.15 (0.08 – 0.26)	0.14 (0.08 – 0.27)	0.16	0.64 (0.32 – 1.00)	<LOQ	<LOQ	<LOQ	<LOQ	<LOQ	<LOQ	<LOQ	<LOQ	<LOQ	0.003 (0.001 – 0.006)	<LOQ	<LOQ
**MePFOSAA**	<LOQ	<LOQ	<LOQ	<LOQ	<LOQ	1.05 (<LOQ – 1.67)	<LOQ (<LOQ – 1.30)	1.23	1.30 (<LOQ – 1.34)	0.91 (<LOQ – 1.30)	1.29	<LOQ	<LOQ	<LOQ	0.60 (<LOQ – 1.40)	<LOQ
**MePFOSAA$_{linear+branched}$**	<LOQ	<LOQ	<LOQ	<LOQ	<LOQ	1.05 (<LOQ – 1.67)	<LOQ (<LOQ – 1.31)	1.32	1.30 (<LOQ – 1.34)	0.91 (<LOQ – 1.51)	1.32	<LOQ	<LOQ	<LOQ	0.78 (0.32 – 1.85)	<LOQ
**EtPFOSAA**	<LOQ	<LOQ	<LOQ	<LOQ	<LOQ	<LOQ	<LOQ	<LOQ	<LOQ	<LOQ	<LOQ	<LOQ	<LOQ	<LOQ	0.99 (0.59 – 2.10)	<LOQ
**EtPFOSAA$_{linear+branched}$**	<LOQ	<LOQ	<LOQ	<LOQ	<LOQ	<LOQ	<LOQ	<LOQ	<LOQ	<LOQ	<LOQ	<LOQ	<LOQ	<LOQ	1.20 (0.66 – 2.50)	<LOQ
**6:2 FTS**	<LOQ	<LOQ	<LOQ	<LOQ	<LOQ	<LOQ	<LOQ	<LOQ	<LOQ	<LOQ	<LOQ	<LOQ	<LOQ	<LOQ	4.60 (<LOQ – 9.85)	<LOQ

**6:2 diPAP**	<LOQ	<LOQ	<LOQ	<LOQ	<LOQ	<LOQ	<LOQ	<LOQ	<LOQ	<LOQ	<LOQ	<LOQ	<LOQ	<LOQ	<LOQ	<LOQ

Table S10: Detection frequencies of the PFAS in the different matrices analysed expressed in % (VG= soil vegetable garden, CE = soil chicken enclosure, GH = soil greenhouse, C = compost, egg = chicken eggs, SF = small fruits, TF = tree fruits, LV = leafy vegetables, SV = stem vegetables, FV = fruiting vegetables, P = pods, RV = rooting vegetables, N = nuts, RW = rainwater, HD = house dust, S = serum).

	**VG (n = 62)**	**CE (n = 38)**	**GH (n = 10)**	**C (n = 36)**	**Egg (n = 37)**	**SF (n = 29)**	**TF (n = 33)**	**LV (n = 8)**	**SV (n = 17)**	**FV (n = 22)**	**P (n = 6)**	**RV (n = 6)**	**N (n = 7)**	**RW (n = 54)**	**HD (n = 129)**	**S (n = 301)**
**PFBA**	89	64	0	100	24	24	33	25	6	9	83	0	14	59	85	71
**PFPeA**	65	37	100	36	3	59	0	0	6	9	50	0	100	43	76	0
**PFHxA**	76	55	90	33	51	48	64	25	71	41	50	67	29	77	56	1
**PFHpA**	68	50	70	11	3	0	9	25	12	9	67	17	0	79	77	21
**PFOA**	100	100	100	81	76	69	61	88	65	55	33	33	71	94	92	100
**PFOA$_{linear+branched}$**	100	100	100	81	76	69	61	88	65	55	33	33	71	98	92	100
**PFNA**	87	71	80	11	57	10	9	12	18	14	17	17	0	57	90	99
**PFDA**	87	84	80	19	100	97	97	100	100	91	100	100	100	56	94	72
**PFUnDA**	45	37	40	6	51	31	61	25	41	32	50	83	86	4	60	10
**PFDoDA**	48	32	30	8	81	72	76	75	76	64	50	83	100	3	46	2
**PFTrDA**	5	11	0	0	65	31	27	38	29	23	50	17	0	0	71	0
**PFTeDA**	15	8	0	6	70	3	0	0	6	0	0	0	0	0	96	0
**PFHxDA**	0	0	0	0	16	59	85	75	88	77	83	83	86	0	75	0
**PFBS**	90	89	100	100	76	66	52	50	35	41	33	50	14	59	94	1
**PFHxS**	24	37	50	6	8	0	0	12	0	0	0	0	0	11	95	100
**PFHxS$_{linear+branched}$**	34	45	60	6	8	0	0	12	0	0	0	0	0	11	96	100
**PFHpS**	0	3	0	0	3	0	0	0	0	0	0	0	0	0	64	12
**PFOS**	100	100	100	97	86	17	12	62	41	9	17	67	14	58	89	100
**PFOS$_{linear+branched}$**	100	100	100	100	86	17	12	62	41	9	17	67	14	64	93	100
**PFBSA**	94	97	100	83	0	0	0	0	0	5	0	0	0	76	23	0
**MePFOSAA**	0	3	0	0	24	69	42	62	59	59	67	50	14	11	75	0.7
**MePFOSAA$_{linear+branched}$**	0	3	0	0	24	69	42	62	59	59	67	50	14	13	81	0.7
**EtPFOSAA**	13	18	20	0	14	3	3	12	6	14	17	17	0	8	86	0
**EtPFOSAA$_{linear+branched}$**	15	18	20	0	14	3	3	12	6	14	17	17	0	9	88	0
**6:2 FTS**	6	13	0	0	14	0	21	12	12	14	50	17	14	24	61	0.3

6:2 diPAP	5	11	0	0	0	0	0	0	0	0	0	0	0	0	0	0

Table S11: Significant soil characteristics for PFAS concentrations in soil vegetable garden (VG) and soil chicken enclosure (CE). Here, the estimates represent the geometric mean ratio (GMR; for continuous PFAS) or odds ratio (OR; for binary PFAS) for each category compared to the reference category (the category with GMR or OR = 1).

PFAS	Soil characteristic	GMR/OR	95% Confidence interval	p-val1[a]	p-val2[b]	p-val2 FDR corrected	R^2 (coefficient of determination)	N
PFBA (VG)	Lutum content	2.67	1.74 – 4.09	<0.0001	<0.0001	**0.001**	0.321	45
PFPeA (VG)	TOC	1.11	1.03 – 1.20	0.009	0.009	0.051	0.111	61
	Lutum content	1.59	1.29 – 1.96	<0.0001	<0.0001	**0.001**	0.244	61
PFHxA (VG)	TOC	1.13	1.06 – 1.20	0.0005	0.001	**0.004**	0.185	61
	Lutum content	1.41	1.17 – 1.71	0.0006	0.001	**0.004**	0.182	61
PFHpA (VG)	TOC	1.13	1.06 – 1.20	0.0002	0.0002	**0.002**	0.207	61
	Lutum content	1.34	1.11 – 1.61	0.003	0.003	**0.018**	0.140	61
$PFOA_{linear}$ (VG)	Lutum content	1.22	1.06 – 0.10	0.007	0.007	0.080	0.118	61
$PFOA_{linear+branched}$ (VG)	TOC	1.07	1.01 – 1.12	0.014	0.014	0.081	0.099	61
	Lutum content	1.23	1.07 – 1.41	0.006	0.006	0.073	0.121	61
PFBS (VG)	Dry matter	0.675	0.509 – 0.897	0.009	0.009	**0.034**	0.111	61
	TOC	1.17	1.07 – 1.29	0.001	0.001	**0.008**	0.160	61
	Lutum content	1.92	1.50 – 2.46	<0.0001	<0.0001	**0.001**	0.313	61
$PFOS_{linear}$ (VG)	pH	0.790	0.646 – 0.967	0.026	0.026	0.311	0.081	61
$PFOS_{linear+branched}$ (VG)	pH	0.802	0.659 – 0.976	0.032	0.032	0.382	0.076	61
PFBSA (VG)	TOC	1.12	1.02 – 1.22	0.019	0.019	0.113	0.090	61
	Lutum content	1.72	1.35 – 2.18	<0.0001	<0.0001	**0.001**	0.250	61
$PFDoDA_{binary}$ (VG)	TOC	1.10	1.00 – 1.20	0.042	0.042	0.250	0.144	61
$PFOA_{linear}$ (CE)	TOC	1.21	1.01 – 1.45	0.043	0.043	0.170	0.109	38
$PFOA_{linear+branched}$ (CE)	TOC	1.22	1.01 – 1.46	0.041	0.041	0.166	0.111	38

PFNA (CE)	pH	0.614	0.438 – 0.859	0.007	0.007	**0.015**	0.183	38
	TOC	1.70	1.29 – 2.23	0.0006	0.001	**0.002**	0.285	38
PFDA (CE)	pH	0.549	0.353 – 0.856	0.012	0.012	**0.024**	0.163	38
	TOC	1.76	1.21 – 2.57	0.006	0.006	**0.024**	0.193	38
$PFOS_{linear}$ (CE)	pH	0.734	0.559 – 0.964	0.032	0.032	0.130	0.121	38
$PFOS_{linear+branched}$ (CE)	pH	0.746	0.570 – 0.976	0.040	0.040	0.158	0.112	38
PFBSA (CE)	pH	0.618	0.423 – 0.903	0.018	0.018	0.070	0.147	38
$PFHpA_{binary}$ (CE)	TOC	1.18	1.02 – 1.37	0.024	0.024	0.096	0.155	38
$PFunDA_{binary}$ (CE)	pH	0.785	0.646 – 0.954	0.015	0.015	**0.030**	0.173	38
	TOC	1.20	1.04 – 1.39	0.015	0.015	**0.030**	0.178	38
$PFDoDA_{binary}$ (CE)	TOC	1.18	1.02 – 1.36	0.025	0.025	0.100	0.145	38

[a] Category-level *p*-value
[b] Overall *p*-value

Table S12: Significant determinants for PFAS concentrations in soil vegetable garden (VG), soil chicken enclosure (CE), and compost (C). Here, the estimates represent the geometric mean ratio (GMR; for continuous PFAS) or odds ratio (OR; for binary PFAS) for each category compared to the reference category (the category with GMR or OR = 1).

**PFAS**	**Determinant**		**GMR/OR**	**95% Confidence interval**	**p-val1[a]**	**p-val2[b]**	**p-val2 FDR corrected**	**R^2 (coefficient of determination)**	**N**
$PFDoDA_{binary}$ (VG)	Composting weeds	Never	1	/	/	0.027	0.250	0.083	61
		Regularly/always	1.91	1.08 – 3.38	0.027				
PFBS (VG)	Composting egg shells	Never	1	/	/	0.020	0.061	0.088	61
		Regularly/always	2.12	1.14 – 3.91	0.020				

[a] Category-level *p*-value
[b] Overall *p*-value

Table S13: Significant determinants for PFAS concentrations in tree fruits (TF) and fruiting vegetables (FV). The estimates represent the geometric mean ratio (GMR; for continuous PFAS) or odds ratio (OR; for binary PFAS) for each category compared to the reference category (the category with GMR or OR=1).

PFAS	Determinant		GMR/OR	95% Confidence interval	p-val1[a]	p-val2[b]	p-val2 FDR corrected	R^2 (coefficient of determination)	N
$PFHxA_{binary}$ (TF)	Type of water device used	Garden hose	1	/	/	0.032	0.225	0.149	31
		Plastic watering can	2.37	1.08 – 5.20	0.032				
PFTrDA (TF)	Type of water device used	Garden hose	1	/	/	0.047	0.328	0.129	31
		Plastic watering can	1.76	1.03 – 3.00	0.047				
PFNA (TF)	Composting pruning waste	Never	1	/	/	0.035	0.122	0.140	32
		Regularly/always	1.66	1.06 – 2.61	0.035				
	Composting grass	Never	1	/	/	0.006	0.041	0.227	32
		Regularly/always	1.72	1.20 – 2.47	0.006				
PFDoDA (TF)	Composting grass	Never	1	/	/	0.013	0.053	0.188	32
		Regularly/always	2.09	1.21 – 3.63	0.013				
	Composting weeds	Never	1	/	/	0.015	0.053	0.181	32
		Regularly/always	2.18	1.21 – 3.94	0.015				
PFDoDA (FV)	Type of water device used	Garden hose	1	/	/	0.029	0.132	0.217	22
		Plastic watering can	0.403	0.189 – 0.860	0.029				
$PFOA_{linear, binary}$ (FV)	Composting grass	Never	1	/	/	0.036	0.178	0.240	22
		Regularly/always	3.55	1.09 – 11.6	0.036				
$PFOA_{linear+branched, binary}$ (FV)	Composting grass	Never	1	/	/	0.036	0.178	0.240	22
		Regularly/always	3.55	1.09 – 11.6	0.036				
$PFTrDA_{binary}$ (FV)	Composting grass	Never	1	/	/	0.041	0.103	0.214	22
		Regularly/always	3.61	1.05 – 12.3	0.041				
	Composting weeds	Never	1	/	/	0.023	0.103	0.262	22
		Regularly/always	4.32	1.22 – 15.2	0.023				

[a] *Category-level p*-value

[b] Overall *p*-value

Table S14: Significant determinants for PFAS concentrations in chicken eggs. The estimates represent the geometric mean ratio (GMR; for continuous PFAS) or odds ratio (OR; for binary PFAS) for each category compared to the reference category (the category with GMR or OR=1).

PFAS	Determinant		GMR/OR	95% Confidence interval	p-val1[a]	p-val2[b]	p-val2 FDR corrected	R^2 (coefficient of determination)	N
PFTrDA	Surface area of chicken enclosure	≤ 20 m²	1	/	/	0.024	0.133	0.137	37
		> 20 m²	2.25	1.15 – 4.40	0.024				
PFTeDA	Surface area of chicken enclosure	≤ 20 m²	1	/	/	0.047	0.368	0.108	37
		> 20 m²	2.29	1.04 – 5.04	0.047				
PFTrDA	Shape of chicken enclosure	Rectangular/square	1	/	/	0.046	0.167	0.109	37
		Polygonal	2.31	1.05 – 5.11	0.046				
$MePFOSAA_{linear}$	Chickens are fed commercial feed	Never	1	/	/	0.021	0.231	0.143	37
		Regularly	0.484	0.269 – 0.872	0.021				
$MePFOSAA_{linear+branched}$	Chickens are fed commercial feed	Never	1	/	/	0.024	0.262	0.138	37
		Regularly	0.487	0.268 – 0.884	0.024				

$PFUnDA_{binary}$	Chickens are fed weeds	Never	1	/	/	0.033	0.272	0.129	37
		Regularly	0.433	0.200 – 0.936	0.033				
PFBS	Chickens are fed weeds	Never	1	/	/	0.048	0.526	0.107	37
		Regularly	3.33	1.06 – 10.5	0.048				
$PFOS_{linear}$	Chickens are fed weeds	Never	1	/	/	0.049	0.440	0.106	37
		Regularly	3.09	1.05 – 9.13	0.049				
PFDoDA	Chickens are fed bread	Never	1	/	/	0.016	0.176	0.155	37
		Regularly	0.453	0.245 – 0.836	0.016				
PFTrDA	Chickens are fed bread	Never	1	/	/	0.004	**0.039**	0.219	37
		Regularly	0.376	0.204 – 0.694	0.004				

[a] Category-level *p*-value
[b] Overall *p*-value

Table S15: Significant determinants for PFAS concentrations in house dust. The estimates represent the geometric mean ratio (GMR; for continuous PFAS) or odds ratio (OR; for binary PFAS) for each category compared to the reference category (the category with GMR or OR=1).

**PFAS**	**Determinant**		**GMR/OR**	**95% Confidence interval**	**p-val1[a]**	**p-val2[b]**	**p-val2 FDR corrected**	**R^2 (coefficient of determination)**	**N**
PFBS	Renovations during sampling	No	1	/	/	0.023	0.215	0.042	123
		Yes	2.23	1.13	4.40				
$PFOA_{linear}$		No	1	/	/	0.045	0.355	0.032	125

	Mechanical ventilation in living room	Yes	0.606	0.373 – 0.985	0.045				
$PFOA_{linear+branched}$	Mechanical ventilation in living room	No	1	/	/	0.049	0.372	0.031	125
		Yes	0.614	0.380 – 0.994	0.049				
PFNA	Mechanical ventilation in living room	No	1	/	/	0.026	0.251	0.050	98
		Yes	0.498	0.272 – 0.913	0.026				
PFHpS	Method of ventilation in living room	Manually	1	/	/	0.032	**0.043**	0.055	125
		Grills	1.02	0.639 – 1.61	0.947				
		Mechanically	0.513	0.311 – 0.847	0.010				
$PFOS_{linear+branched}$	Method of ventilation in living room	Manually	1	/	/	0.048	0.150	0.049	123
		Grills	0.938	0.653 – 1.35	0.730				
		Mechanically	0.612	0.416 – 0.901	0.014				
$EtPFOSAA_{linear}$	Method of ventilation in living room	Manually	1	/	/	0.009	0.067	0.106	87
		Grills	1.37	0.769 – 2.43	0.290				
		Mechanically	0.387	0.195 – 0.767	0.008				
$EtPFOSAA_{linear+branched}$	Method of ventilation in living room	Manually	1	/	/	0.011	0.074	0.101	88
		Grills	1.34	0.749 – 2.40	0.326				
		Mechanically	0.386	0.192 – 0.773	0.009				
PFHpS	Method of ventilation in bedroom	Manually	1	/	/	0.027	0.100	0.057	126
		Grills	1.04	0.574 – 1.88	0.902				
		Mechanically	0.507	0.309 – 0.833	0.008				
$EtPFOSAA_{linear}$		Manually	1	/	/	0.013	0.081	0.097	88

	Method of ventilation in bedroom	Grills	2.01	0.984 – 4.12	0.059				
		Mechanically	0.500	0.258 – 0.970	0.043				
$EtPFOSAA_{linear+branched}$	Method of ventilation in bedroom	Manually	1	/	/	0.016	0.077	0.091	89
		Grills	2.01	0.972 – 4.17	0.063				
		Mechanically	0.508	0.259 – 0.996	0.052				
PFHpS	Mechanical ventilation in living room or bedroom	No	1	/	/	0.005	**0.043**	0.061	125
		Yes	0.515	0.325 – 0.815	0.005				
$PFOS_{linear}$	Mechanical ventilation in living room or bedroom	No	1	/	/	0.032	0.204	0.037	123
		Yes	0.673	0.471 – 0.963	0.032				
$PFOS_{linear+branched}$	Mechanical ventilation in living room or bedroom	No	1	/	/	0.025	0.108	0.041	123
		Yes	0.661	0.462 – 0.944	0.025				
$EtPFOSAA_{linear}$	Mechanical ventilation in living room or bedroom	No	1	/	/	0.033	0.089	0.053	87
		Yes	0.497	0.264 – 0.934	0.033				
$EtPFOSAA_{linear+branched}$	Mechanical ventilation in living room or bedroom	No	1	/	/	0.039	0.107	0.049	88
		Yes	0.505	0.266 – 0.957	0.039				
$PFBSA_{binary}$		Never	1	/	/	0.004	0.084	0.135	96

	Frequency of ventilation in bedroom	Daily (short)	0.975	0.391 – 2.43	0.956				
		Daily (long)	4.02	1.71 – 9.45	0.001				
6:2 FTS	Frequency of ventilation in bedroom	Never	1	/	/	0.025	0.159	0.059	125
		Daily (short)	0.562	0.350 – 0.903	0.019				
		Daily (long)	0.529	0.327 – 0.855	0.011				
6:2 FTS	Frequency of sweeping living room	≤ Weekly	1	/	/	0.021	0.159	0.043	125
		> Weekly	0.576	0.363 – 0.914	0.021				
PFPeA	Frequency of cleaning living room with water	≤ Weekly	1	/	/	0.045	0.533	0.037	110
		> Weekly	1.45	1.02 – 2.08	0.045				
PFHpS	Frequency of sweeping bedroom	≤ Weekly	1	/	/	0.045	0.121	0.032	126
		> Weekly	0.554	0.313 – 0.980	0.045				
$EtPFOSAA_{linear}$	Frequency of dusting bedroom	Never	1	/	/	0.045	0.122	0.070	88
		Monthly	1.02	0.569 – 1.84	0.938				
		> Monthly	1.90	1.12 – 3.23	0.019				
$EtPFOSAA_{linear+branched}$	Frequency of dusting bedroom	Never	1	/	/	0.040	0.110	0.072	89
		Monthly	0.947	0.523 – 1.72	0.857				
		> Monthly	1.87	1.10 – 3.18	0.023				
PFTeDA	Room in which dust sample was taken	Living room	1	/	/	0.009	0.089	0.103	109
		Bedroom	0.487	0.308 – 0.769	0.003				
		Both	0.476	0.273 – 0.831	0.010				
		Other	0.589	0.305 – 1.14	0.119				
PFBS		Living room	1	/	/	0.009	0.181	0.097	116

	Room in which dust sample was taken	Bedroom	0.514	0.320 – 0.825	0.007				
		Both	0.516	0.287 – 0.929	0.030				
		Other	0.379	0.187 – 0.771	0.009				
$PFHxS_{linear}$	House dust scraped from top of cabinet	No	1	/	/	0.014	0.264	0.050	120
		Yes	1.76	1.13 – 2.75	0.014				
$PFHxS_{linear+branched}$	House dust scraped from top of cabinet	No	1	/	/	0.021	0.225	0.044	120
		Yes	1.71	1.09 – 2.67	0.021				
PFHpS	House dust scraped from top of cabinet	No	1	/	/	0.031	0.100	0.039	120
		Yes	1.59	1.05 – 2.40	0.031				
$PFOS_{linear}$	House dust scraped from top of cabinet	No	1	/	/	0.004	0.070	0.070	118
		Yes	1.61	1.18 – 2.22	0.004				
$PFOS_{linear+branched}$	House dust scraped from top of cabinet	No	1	/	/	0.005	0.089	0.067	118
		Yes	1.59	1.16 – 2.19	0.005				
$MePFOSAA_{linear}$	House dust scraped from top of cabinet	No	1	/	/	0.007	0.141	0.109	65
		Yes	2.28	1.27 – 4.09	0.007				
$MePFOSAA_{linear+branched}$	House dust scraped	No	1	/	/	0.023	0.441	0.079	65

	from top of cabinet	Yes	2.01	1.12 – 3.63	0.023				
$EtPFOSAA_{linear}$	House dust scraped from top of cabinet	No	1	/	/	0.019	0.089	0.064	85
		Yes	1.99	1.13 – 3.48	0.019				
$EtPFOSAA_{linear+branched}$	House dust scraped from top of cabinet	No	1	/	/	0.015	0.077	0.069	85
		Yes	2.06	1.16 – 3.65	0.015				
$PFDoDA_{binary}$	Last time that the cabinet was cleaned	> 1 year ago	1	/	/	0.007	0.137	0.113	88
		> 3 months ago	0.529	0.274 – 1.02	0.058				
		< 3 months ago	0.793	0.322 – 1.95	0.615				
PFNA	Last time the cabinet was cleaned	> 1 year ago	1	/	/	0.004	0.072	0.172	62
		> 3 months ago	0.572	0.376 – 0.871	0.012				
		< 3 months ago	0.337	0.166 – 0.684	0.004				
PFDA	Last time the cabinet was cleaned	> 1 year ago	1	/	/	0.017	0.314	0.115	70
		> 3 months ago	0.502	0.312 – 0.807	0.006				
		< 3 months ago	0.537	0.265 – 1.09	0.088				
PFUnDA	Last time the cabinet was cleaned	> 1 year ago	1	/	/	0.037	0.513	0.089	74
		> 3 months ago	0.679	0.452 – 1.02	0.067				
		< 3 months ago	0.478	0.259 – 0.884	0.022				
PFTrDA		> 1 year ago	1	/	/	0.003	0.065	0.125	88

	Last time the cabinet was cleaned	> 3 months ago	0.624	0.442 – 0.882	0.009				
		< 3 months ago	0.441	0.257 – 0.757	0.004				
PFTeDA	Last time the cabinet was cleaned	> 1 year ago	1	/	/	0.005	0.089	0.128	81
		> 3 months ago	0.484	0.313 – 0.751	0.002				
		< 3 months ago	0.511	0.264 – 0.989	0.050				
$PFHxS_{linear+branched}$	Last time the cabinet was cleaned	> 1 year ago	1	/	/	0.024	0.225	0.084	88
		> 3 months ago	0.533	0.342 – 0.831	0.007				
		< 3 months ago	0.647	0.323 – 1.30	0.224				

[a] Category-level *p*-value

[b] Overall *p*-value

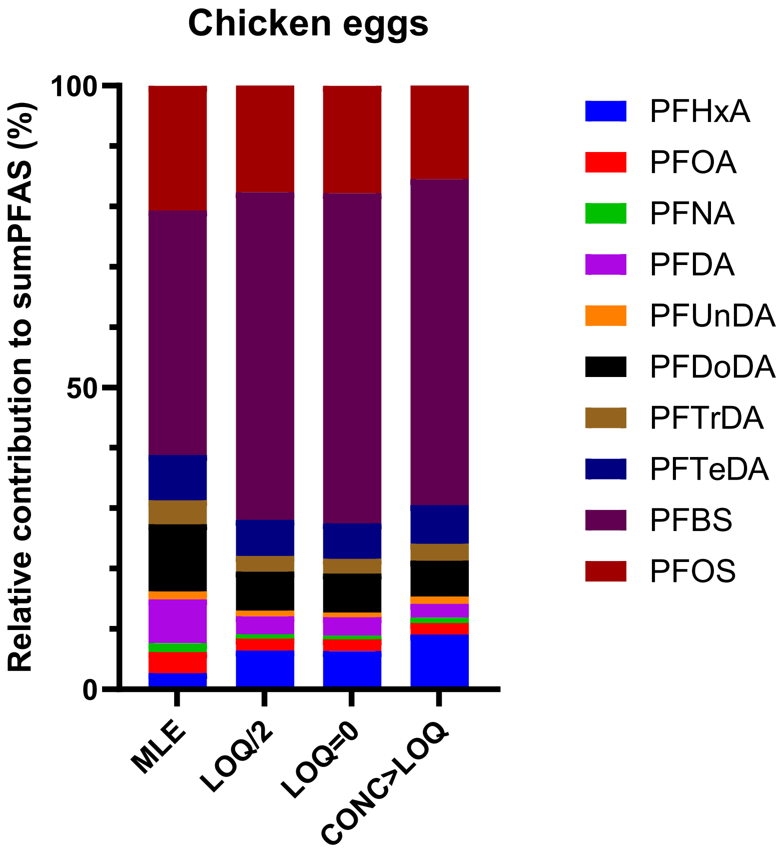

Figure S1: Side-by-side comparison of PFAS compositional profiles of chicken eggs obtained using four different substitution methods (MLE substitution, substitution by LOQ/2, substitution by LOQ = 0, and using only samples with concentrations >LOQ), with PFAS that were detected <50% in the samples omitted in all cases. The figures show strong similarity and support the robustness of the MLE substitution approach.

ASTM 2019. Standard Test Methods for Laboratory Determination of Water (Moisture) Content of Soil and Rock by Mass. *Book of Standards Volume: 04.08.*
ISO 2020. Determination of particle size distribution in mineral soil material: Method by sieving and sedimentation.
ISO 2021. Sludge, treated biowaste and soil—Determination of pH.
NBN 2022. Soil, waste, treated biowaste and sludge—Determination of total organic carbon (TOC) by dry combustion.
RDC 2022. Regulations on the analysis of microdata. Düsseldorf, Germany: Research Data Centres of the Federal Statistical Office and the Statistical Offices of the Federal States (RDC).
